# Habitat quality influences pollinator pathogen prevalence through both habitat–disease and biodiversity–disease pathways

**DOI:** 10.1002/ecy.3933

**Published:** 2023-01-03

**Authors:** Michelle L. Fearon, Chelsea L. Wood, Elizabeth A. Tibbetts

**Affiliations:** ^1^ Department of Ecology & Evolutionary Biology University of Michigan Ann Arbor Michigan USA; ^2^ School of Aquatic and Fishery Sciences University of Washington Seattle Washington USA

**Keywords:** black queen cell virus, *Bombus impatiens*, deformed wing virus, dilution effect, floral resources, honey bees, multihost–multiparasite, native bees, sacbrood virus, structural equation models

## Abstract

The dilution effect hypothesis posits that increasing biodiversity reduces infectious disease transmission. Here, we propose that habitat quality might modulate this negative biodiversity–disease relationship. Habitat may influence pathogen prevalence directly by affecting host traits like nutrition and immune response (we coined the term “habitat–disease relationship” to describe this phenomenon) or indirectly by changing host biodiversity (biodiversity–disease relationship). We used a path model to test the relative strength of links between habitat, biodiversity, and pathogen prevalence in a pollinator–virus system. High‐quality habitat metrics were directly associated with viral prevalence, providing evidence for a habitat–disease relationship. However, the strength and direction of specific habitat effects on viral prevalence varied based on the characteristics of the habitat, host, and pathogen. In general, more natural area and richness of land‐cover types were directly associated with increased viral prevalence, whereas greater floral density was associated with reduced viral prevalence. More natural habitat was also indirectly associated with reduced prevalence of two key viruses (black queen cell virus and deformed wing virus) via increased pollinator species richness, providing evidence for a habitat‐mediated dilution effect on viral prevalence. Biodiversity–disease relationships varied across viruses, with the prevalence of sacbrood virus not being associated with any habitat quality or pollinator community metrics. Across all viruses and hosts, habitat–disease and biodiversity–disease paths had effects of similar magnitude on viral prevalence. Therefore, habitat quality is a key driver of variation in pathogen prevalence among communities via both direct habitat–disease and indirect biodiversity–disease pathways, though the specific patterns varied among different viruses and host species. Critically, habitat–disease relationships could either contribute to or obscure dilution effects in natural systems depending on the relative strength and direction of the habitat–disease and biodiversity–disease pathways in that host–pathogen system. Therefore, habitat may be an important driver in the complex interactions between hosts and pathogens.

## INTRODUCTION

From its inception in the early 2000s, demonstrations of the dilution effect hypothesis have depended on some habitats being better for biodiversity than others (Ostfeld & Keesing, 2000). The dilution effect posits that increasing biodiversity decreases infectious disease transmission because added species regulate the density of competent hosts or absorb infectious propagules that would otherwise pass to competent hosts (Keesing et al., 2006). The earliest dilution effect studies varied habitat quality (e.g., forest fragmentation or urbanization) to obtain replicates that varied in their levels of biodiversity (Allan et al., 2003; Ezenwa et al., 2006; Ostfeld & Keesing, 2000). Despite the link between habitat quality and biodiversity, no studies to date have discriminated between two plausible mechanisms that might explain apparent dilution effects: (1) increasing habitat quality increases biodiversity and biodiversity reduces disease or (2) increasing habitat quality has direct effects on infectious disease transmission and increases biodiversity, but biodiversity does not directly influence infectious disease transmission. These two possibilities are not mutually exclusive. However, in extreme cases, the relationship between biodiversity and disease may be due to a common correlation with habitat rather than a causal relationship where biodiversity reduces disease transmission.

Habitat characteristics could directly impact pathogen prevalence through effects on host nutrition or immune response to pathogens, a hypothesis that we call the “habitat–disease relationship” to parallel the “biodiversity–disease” relationship proposed by the dilution effect hypothesis. The habitat–disease relationship is plausible because an animal's habitat determines the quantity and quality of resources available and the nutritional status of residents (Donkersley et al., 2014; Wilkin et al., 2009), which can modulate immune responses to infection (Smith, 2007). In many host–pathogen systems, poor nutrition decreases host immune function and increases host susceptibility and/or disease burdens (Ponton et al., 2013; Smith, 2007). For instance, the habitat and nutritional status of Eurasian red squirrels (*Sciurus vulgaris*) influence the abundance of their host‐specific, directly transmitted gastrointestinal helminth parasites. Squirrels in fragmented habitats host greater parasite burdens than do those in continuous forest habitats, and parasite burdens were higher in years with low food availability (Santicchia et al., 2015). In addition to satisfying nutritional requirements, resources might also have medicinal qualities that directly reduce infections (Richardson et al., 2015). Of course, the ways in which habitat characteristics influence pathogens are likely to vary based on the specific ecology of the hosts and pathogens involved. For example, some pathogens may thrive in “good” habitats because they can exploit additional host resources (Penczykowski et al., 2014). Overall, there is strong evidence in many systems that habitat factors directly influence host nutrition and immunocompetence, which may positively or negatively impact pathogen replication and transmission.

We present the habitat–disease relationship as an alternative, non‐mutually‐exclusive explanation for previously observed biodiversity–disease relationships. Habitat characteristics that improve host nutrition are often also linked with high biodiversity (Foley et al., 2005). Consequently, habitat–disease and biodiversity–disease pathways may work in concert to reduce pathogen prevalence in hosts from high‐quality habitats that live in species‐rich communities. Some apparent relationships between biodiversity and pathogen prevalence could be caused, in part, by a spurious correlation in which the causal driver is in fact habitat. Alternatively, the habitat and biodiversity pathways may work in opposition, canceling out any apparent correlation between habitat quality, species richness, and pathogen prevalence. Habitat–disease relationships represent a previously unexplored mechanism that could plausibly drive variable patterns of pathogen prevalence among communities and help explain why, although dilution is often observed (Civitello et al., 2015), we still cannot explain its idiosyncratic appearance across systems (Salkeld et al., 2013; Wood et al., 2014) and scales (Wood et al., 2017).

In this study, we tested whether habitat characteristics directly mediated pathogen prevalence among multiple hosts in a host–pathogen system with previous evidence of the dilution effect (Fearon & Tibbetts, 2021). Our previous study did not include information about how habitat influences biodiversity or pathogen prevalence. The goal of the current study was to determine both the direct and indirect effects of habitat on pathogen prevalence to better understand the mechanisms underlying variable pathogen prevalence. We worked in a pollinator system with multiple bee hosts and viruses, including black queen cell virus (BQCV), deformed wing virus (DWV), and sacbrood virus (SBV). Pollinators are ideal for exploring habitat–disease relationships because better habitat improves pollinator nutrition, and pollinator nutrition influences immunocompetence and infection. In particular, pollinator foraging patterns and nutrition are tightly linked with access to locally abundant and diverse flowers and greater natural grassland and woodland area (Donkersley et al., 2014; Jha & Kremen, 2013). Additionally, bees that consume diverse and high‐protein pollen diets have improved immunocompetence (Alaux et al., 2010; Brunner et al., 2014), reduced parasite infections (DeGrandi‐Hoffman et al., 2010; Di Pasquale et al., 2013), and reduced infection‐caused mortality (Dolezal et al., 2019). We define high‐quality habitats as areas that provide sufficient quantity and diversity of floral resources to sustain good pollinator nutrition. Therefore, we used high local floral richness and density and the high landscape‐level proportion of natural area and landscape richness (a proxy of floral diversity based on diversity of land‐cover types) as the key high‐quality habitat characteristics to test for the habitat–disease relationship.

Though previous field studies investigating the effects of habitat characteristics on pollinator health have been rare, a few recent studies have shown interesting, yet complex, links among habitat metrics and pollinator pathogen prevalence. Greater floral abundance correlated with reduced bumblebee BQCV and DWV loads, and immune gene expression increased in areas with greater grassland cover (McNeil et al., 2020). Simplified agricultural landscapes increases bee diet breadth and, consequently, dilutes parasite prevalence in bee communities (Figueroa et al., 2020). Additionally, in monoculture environments, native bees have greater parasite prevalence when noncrop flower abundance is low, but as noncrop flower abundance increases, the relationship flips to reduce parasite prevalence (Cohen et al., 2021). These studies showed that habitat–disease pathways were likely to have important effects on patterns of pollinator pathogen prevalence but may vary among different kinds of habitat characteristics.

In addition to mediating pollinator health, many habitat characteristics simultaneously influence pollinator community diversity and abundance, with apparent dilution effects on pathogen prevalence. Increasing the size and diversity of floral patches, landscape heterogeneity, and natural area increases pollinator richness and abundance (Blaauw & Isaacs, 2014; Kennedy et al., 2013). Our previous research showed that three pollinator viruses, BQCV, DWV, and SBV, exhibited “diluted” viral prevalence in species‐rich pollinator communities compared to species‐poor communities for multiple bee hosts (Fearon & Tibbetts, 2021). However, it remains unclear whether this pattern is driven by high‐quality habitat characteristics, pollinator species richness, or a combination of both pathways.

Disentangling the relative impact of habitat quality through direct habitat–disease relationships and indirect biodiversity–disease relationships is critical to understand the observed variation in viral prevalence among pollinator communities. We surveyed BQCV, DWV, and SBV prevalence in pollinator communities with variable local and landscape habitat characteristics and employed path models to address three main questions. First, are local‐ and landscape‐scale habitat characteristics directly linked with pathogen prevalence? Second, are local‐ and landscape‐scale habitat characteristics indirectly associated with pathogen prevalence through habitat‐mediated changes in pollinator community diversity and/or abundance? Finally, do direct habitat–disease and indirect biodiversity–disease pathways have similar relative strengths and directions? We used path models to evaluate the relative magnitude and direction of all direct and indirect pathways between habitat characteristics and viral prevalence, while accounting for the effects of all other significant pathways. We first constructed a path model that included combined virus prevalence within the four most common pollinator species in the communities to understand the drivers of community‐wide changes in viral prevalence. Then we generated host species‐specific path models for *Apis mellifera*, *Bombus impatiens*, *Lasioglossum* spp., and *Eucera pruinosa* to evaluate whether habitat quality and pollinator community factors impacted host species and/or viruses differently. Overall, these path models allow us to rigorously disentangle the effects of habitat characteristics on patterns of pathogen prevalence through the newly proposed habitat–disease relationship and the well‐established biodiversity–disease relationship.

## METHODS

The pollinator and viral prevalence sampling and data were previously included in Fearon and Tibbetts (2021), which demonstrated the dilution effect for BQCV, DWV, and SBV in multiple host species. The current study focuses on the mechanisms driving the previously observed patterns and includes new data on local‐ and landscape‐scale habitat.

### Pollinator community sampling

We sampled pollinator communities at 14 winter squash farms in southeastern Michigan, USA, with private landowner permission, as previously described in Fearon and Tibbetts (2021) (Appendix S1: Table S1). The field sites were surrounded by a landscape gradient of monoculture agriculture to natural forests (6%–88% natural area in a 1000‐m radius). Field sites were situated 10 km or more from one another, so it was unlikely that bees would be able to visit more than one field site (Greenleaf et al., 2007). We visited each site twice during the peak squash flower bloom: five sites between 22 July and 21 August 2015 and eight sites between 26 July and 2 September 2016.

Pollinators were collected with hand‐netting and pan traps along four 50‐m transects. Three transects were randomly placed in the field along crop rows, and one was placed along the field edge containing native and invasive flowering plants. Sampling effort of the pollinator communities was standardized in terms of both total time and area sampled: We netted all pollinators observed for 30 min within 1.5 m of the transect line. Each transect was sampled by hand net once between 8:00 a.m. and 12:00 p.m. Pan traps were set 5 m apart along each transect for 6 h. Pollinator collection ceased by 1:00 p.m. because pollinator activity declined after squash flowers closed at midday. All collected insects were stored on dry ice in the field and then placed in a −80°C freezer in the lab.

All 4330 specimens were identified using the Discover Life key (Ascher & Pickering, 2020). We identified most specimens to species, though some groups were only identified to genus because they are difficult to key out (e.g., *Lasioglossum* spp.). The pollinator communities were highly variable in species richness (seven to 49 species) and abundance (46 to 756 individuals). The most abundant species were *Lasioglossum* spp. (*n* = 1218), *B. impatiens* (*n* = 1045), *E. pruinosa* (*n* = 557), and *A. mellifera* (*n* = 505).

### Habitat quantification at local and landscape scales

We quantified habitat diversity and abundance at local‐ and landscape‐level spatial scales. We sampled local floral species richness and total floral density within 1‐m^2^ plots at 10‐m intervals along the length of each pollinator‐sampling transect (*n* = 48 per site). In each plot, we recorded the number of flowers for each plant species observed. We obtained total floral density by summing the total number of flowers across all plots at a site and dividing by the total area sampled. Floral species richness was quantified as the number of herbaceous flowering plant species observed at a site. One site originally included in Fearon and Tibbetts (2021) was missing local habitat data and was removed from analyses in this manuscript.

We used landscape data obtained from the 2015 and 2016 USDA cropland data layers (USDA National Agricultural Statistics Service Cropland Data Layer,  2015–2016), which classifies the dominant land‐cover type within each 30 × 30‐m grid cell of the USA. We grouped deciduous, evergreen, and mixed forests, herbaceous and woody wetlands, shrubland, grass pasture, and wildflower meadow land‐cover types into one “natural habitat” category because those land‐cover types provide important foraging resources for many bee species (Koh et al., 2016). A geographic information system (GIS) was used to quantify the proportion of natural habitat surrounding each site at 1000‐m radii. We calculated landscape richness by counting all land‐cover types within the 1000‐m radius to quantify the variety of potential floral resources available to pollinators in the surrounding landscape (full list of land‐cover types in Appendix S1: Table S2). We used a 1000‐m spatial scale for all analyses because most pollinators forage within a 1000‐m radius (Greenleaf et al., 2007).

### Detecting viral prevalence and active replication

We tested for BQCV, DWV, and SBV prevalence in a subset of collected pollinators with up to 20 randomly selected per species from each site (*A. mellifera*, *n* = 234; *B. impatiens*, *n* = 248; *Lasioglossum* spp., *n* = 233; *E. pruinosa*, *n* = 173; Appendix S1: Table S3). These four species were the most common and therefore allowed for comparison of viral prevalence among all communities. At sites with fewer than 20 individuals from a species, we tested all individuals for viral presence.

The pollinator samples used for viral detection were previously used in Fearon and Tibbetts (2021), where more detailed methods are available. Briefly, we extracted RNA from each bee's gut tissue with TRIzol reagent (Ambion, Austin, TX, USA), and positive strand complementary DNA (cDNA) synthesis reactions were performed with 2 μl of RNA template with M‐MLV reverse transcriptase (Promega, Madison, WI, USA) and 0.25 μM random hexamers (Invitrogen, Carlsbad, CA, USA).

We tested for the presence of BQCV, DWV, and SBV using polymerase chain reaction (PCR) with virus‐specific positive strand primers (Appendix S1: Table S4). The DWV primer did not differentiate between DWV‐A, ‐B, or ‐C variants. Therefore, reported DWV prevalence includes all three variants. As a positive control for RNA extraction and reverse transcription, we tested each sample for the presence of the 18 S rRNA gene (Cardinal et al., 2010). All reactions included negative (H_2_O) and virus‐positive controls. PCR products were visualized with gel electrophoresis to determine the presence or absence of each virus in each specimen.

Our previous work in this system demonstrated that all four host species produced active infections of BQCV, DWV, and SBV (except for SBV in *Lasioglossum* spp.) by testing for the viral negative strand (Fearon & Tibbetts, 2021). A subset of the PCR products from the 18 S, and positive‐ and negative‐strand reactions were sequenced to confirm identification (GenBank Accession Numbers: MN900314–MN900321; MN902093–MN902138).

### Statistical analysis

**FIGURE 1:**
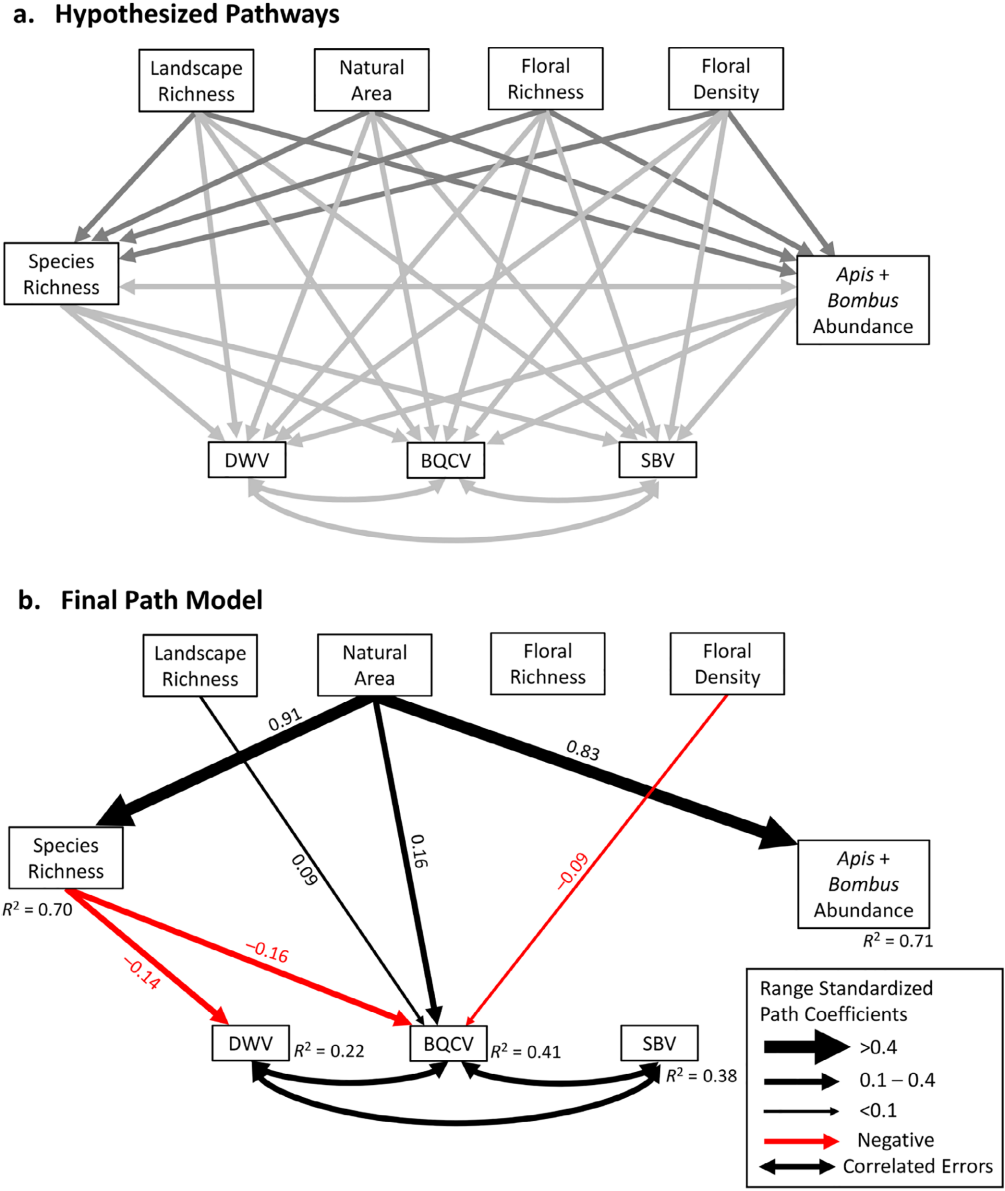
(a) All hypothesized pathways in the initial path model for effects of local‐ and landscape‐level habitat and pollinator community characteristics on black queen cell virus (BQCV), deformed wing virus (DWV), and sacbrood virus (SBV) prevalence within four focal pollinator host species: *Apis mellifera*, *Bombus impatiens*, *Lasioglossum* spp., and *Eucera pruinosa*. Dark gray arrows are modeled with linear regressions; light gray paths are modeled with generalized linear mixed models with binomial distributions. Double‐headed arrows indicate correlated errors included in model. (b) The final path model shows that habitat characteristics are directly linked with BQCV prevalence and indirectly linked with BQCV and DWV prevalence through pollinator species richness, but not combined *A. mellifera* and *B. impatiens* abundance. Significant negative (red) and positive (black) associations between linked variables are shown, but nonsignificant paths are not. Path thickness corresponds to the magnitude of the range standardized regression coefficients, which indicate the proportional shift in the response variable given a full shift in the predictor variable along its range. Full model statistics can be found in Appendix S2: Table S3.

We used path models to examine the relative impact of local‐ and landscape‐level habitat characteristics and pollinator community features on BQCV, DWV, and SBV prevalence in four bee host species: *A. mellifera*, *B. impatiens*, *Lasioglossum* spp., and *E. pruinosa*. In the path model, local‐ and landscape‐scale habitat factors (i.e., floral richness, floral density, landscape richness, and proportion of natural area) could affect pollinator community species richness and pollinator abundance through linear regressions of values for each site (*n* = 13, dark gray lines, Figure 1a). All habitat and community variables could affect BQCV, DWV, and SBV prevalence through generalized linear mixed models with binomial distributions and included random effects for bee host species and the visit number to each site nested within a site (*n* = 888, light gray lines, Figure 1a). The model included correlated errors among the three viruses and between pollinator species richness and abundance. We compared the Akaike information criterion (AIC) of different versions of this main path model that replaced the total pollinator abundance term with either *A. mellifera*, *B. impatiens*, *Lasioglossum* spp., or *E. pruinosa* abundance or combined *A. mellifera* and *B. impatiens* abundance to determine whether the abundance of a particular host species improved the model fit. We found that the combined abundance of *A. mellifera* and *B. impatiens* provided the lowest AIC path model, so this is the model presented in the main text.

All the path models converged and were completely saturated; therefore, we could not assess the causality of the paths in these models or conduct tests of directed separation for the model (all models: χ^2^ = 0, *p* = 1). To better assess the goodness of fit, we temporarily simplified the main path model by removing paths with *p* values >0.8 following Grace (2020). The χ^2^ indicated that the data fit our hypothesized model structure well (i.e., no significant difference between relationships in the data and model structure), and the tests of directed separation showed that we were not missing any important pathways (χ^2^ = 0.044, *p* = 0.978; Fisher's *C* = 0.489, *p* = 0.975, respectively; see Appendix S2: Section S1 for additional details). We calculated the range‐standardized coefficients (RC) for each path, which indicate the proportional shift in the response variable along its range given a full shift in the predictor variable along its range. These values allow for easy interpretation and comparison of the relative effect of each predictor on the response, holding other variables in the model constant (Lefcheck, 2019). We also calculated the scale‐standardized coefficients (SC) for each path, which standardizes each variable by its SD and are expressed in equivalent units, to be able to compare the relative magnitude of change for direct versus indirect pathways within the model, since range‐standardized coefficients are not suitable for such applications due to differences in variable units (Lefcheck, 2019). Further description of the path model details, including variable transformations, correlated errors, goodness‐of‐fit tests, tests of component model assumptions and spatial autocorrelation, AIC comparison table, and all model outputs, is presented in Appendix S2.

Next, we compared the relative effects of significant direct versus indirect pathways between habitat characteristics and viral prevalence. First, we calculated the coefficient for each indirect pathway between habitat characteristics and viral prevalence by multiplying the scale‐standardized coefficient of each component pathway (e.g., natural area → species richness [0.63] × species richness → DWV prevalence [−0.24] = −0.15, Appendix S2: Table S3). For each virus, we determined the net effect of direct and indirect pathways by summing the significant coefficients for each pathway type. Lastly, we calculated the total net effect of all habitat and pollinator community characteristics on prevalence of each virus by summing all significant direct and indirect pathway coefficients for a given virus (nonsignificant paths were not included in any of the net effect calculations).

We also conducted a parallel analysis with estimated species richness to compare with the observed pollinator species richness in the main analysis because it is rare to reach an asymptote when sampling invertebrate communities (Gotelli & Colwell, 2001; Novotný & Basset, 2000). We found that the topography of significant pathways in the estimated richness path model varied slightly from the main path model presented with the lowest AIC but was overall consistent with the results from the other versions of the main path model described earlier. Additionally, the estimated richness path model had a ΔAIC of 9.11 higher than the model with the lowest AIC, so our results were robust to our choice of species richness measurement (details in Appendix S2: Section S2 and Table S11).

Finally, we also ran separate host species‐specific path models to elucidate how habitat and pollinator community characteristics affected BQCV, DWV, and SBV prevalence within each bee host. These models had the same path structure as the main model described earlier but only included viral prevalence data from a single host species. SBV presence was excluded from the *Lasioglossum* spp.‐ and *E. pruinosa*‐specific models because SBV was very rare in those species (Fearon & Tibbetts, 2021). Additionally, the *E. pruinosa*‐specific models only included 12 sites because *E. pruinosa* was not detected at one site (K site). All the species‐specific path models were completely saturated (details in Appendix S3).

## RESULTS

### Are local‐ and landscape‐scale habitat characteristics directly linked with pathogen prevalence?

**FIGURE 2:**
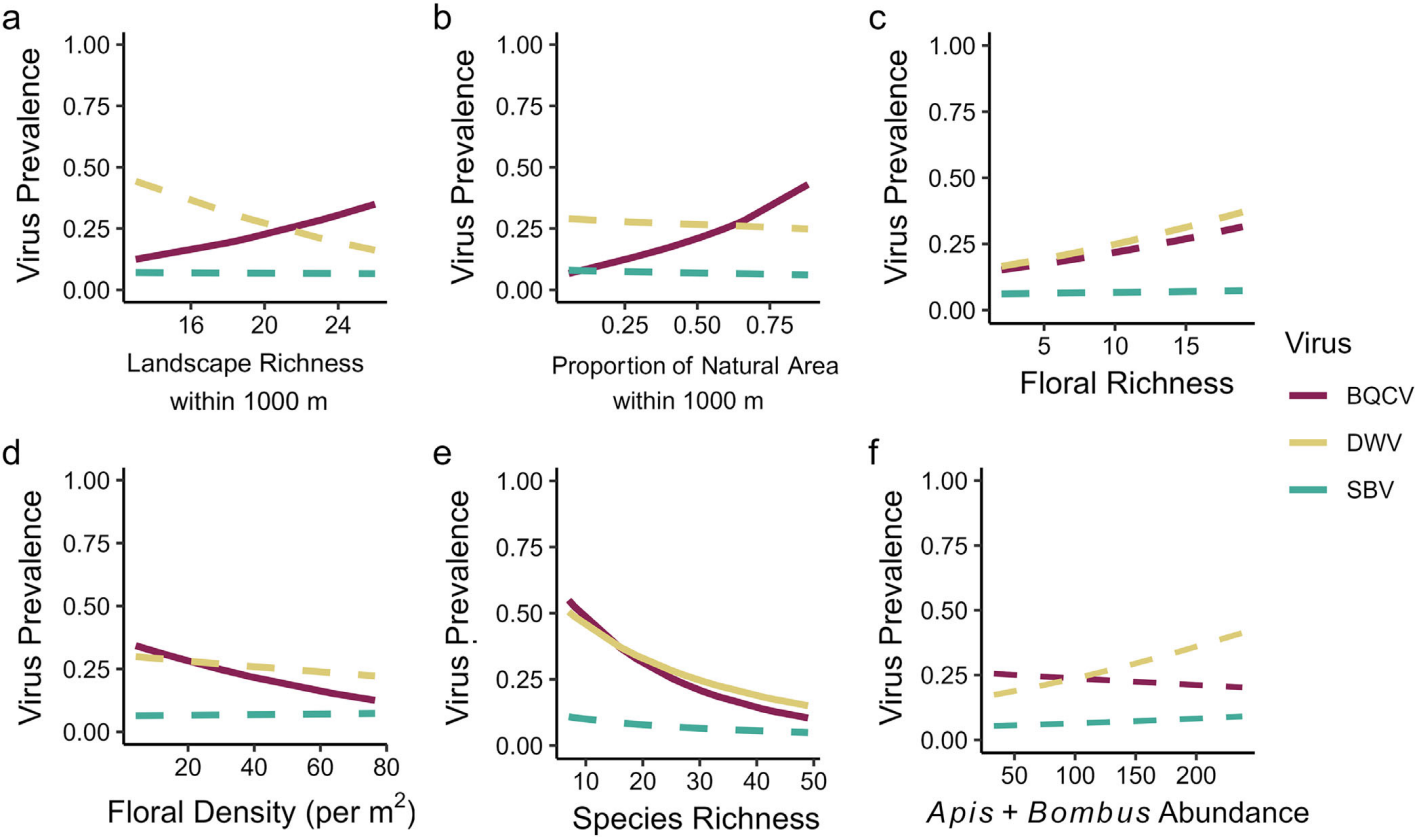
Model‐predicted black queen cell virus (BQCV), deformed wing virus (DWV), and sacbrood virus (SBV) prevalence varies among sites with different (a) landscape richness within 1000 m, (b) proportion natural area within 1000 m, (c) floral richness, (d) floral density, (e) pollinator community species richness, and (f) combined *Apis mellifera* and *Bombus impatiens* abundance, based on component BQCV, DWV, and SBV binomial generalized linear mixed models in the main path model (Appendix S1: Table S3). Significant relationships in the main path model are displayed as solid lines, nonsignificant relationships are dashed. Red = BQCV; yellow = DWV; teal blue = SBV. Overlapping confidence intervals were omitted to improve figure clarity.

Direct links between habitat quality characteristics and viral prevalence varied among the three viruses, and individual habitat–disease pathways varied in the direction of their effects based on the specific habitat factor (Figures 1b and 2a–d). In the path model including all host species, BQCV was significantly associated with several different habitat factors, whereas DWV and SBV prevalence was not linked with any habitat factors (Appendix S2: Table S3). At the landscape scale, greater proportions of natural area (RC = 0.16) and higher landscape richness (RC = 0.09) were directly associated with greater BQCV prevalence. Greater local floral density was the only habitat factor that correlated with reduced BQCV prevalence (RC = −0.09).

**FIGURE 3:**
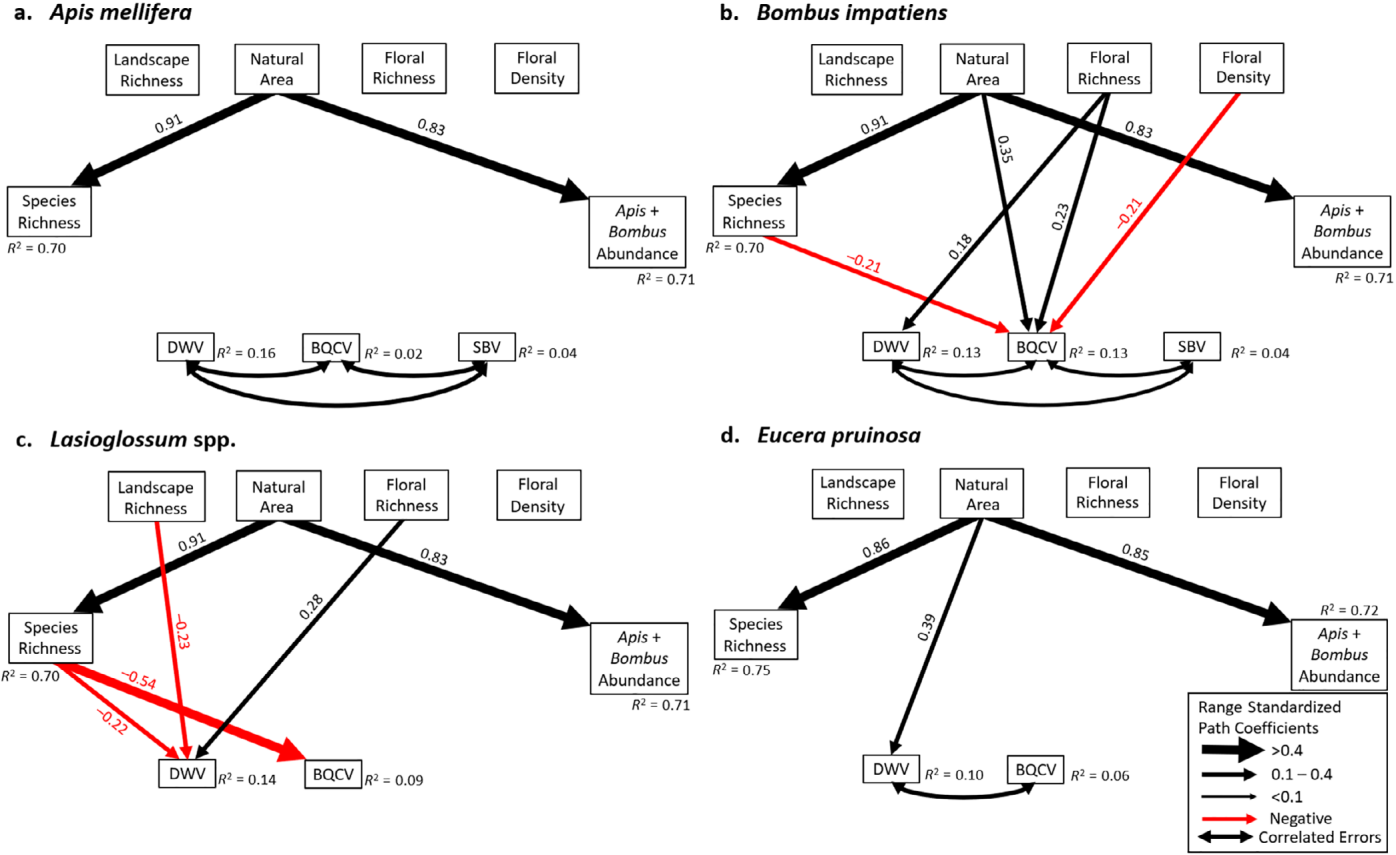
Separate path models including viral prevalence within only (a) *Apis mellifera*, (b) *Bombus impatiens*, (c) *Lasioglossum* spp., and (d) *Eucera pruinosa* hosts, respectively. Sacbrood virus (SBV) is extremely rare in *Lasioglossum* spp. and *E. pruinosa*, so the SBV component model was removed from the path models for those species. Each model included all possible links between habitat characteristics, pollinator community characteristics, and each virus, but only significant paths are shown in the figure. Red and black paths denote significant negative and positive associations between linked variables, respectively, and path thickness corresponds to the magnitude of the range standardized coefficients. Double‐headed arrows indicate correlated errors included in the model. Model statistics can be found in Appendix S3. BQCV, black queen cell virus; DWV, deformed wing virus.

Host species‐specific path models indicated that viral prevalence in each host species was affected by a unique combination of pathways between habitat characteristics and viral prevalence (Figure 3, Appendix S3: Tables S1–S4). Viral prevalence within *A. mellifera* was not associated with any habitat factors, but the other three host species had at least one direct habitat–disease link. In *B. impatiens*, BQCV prevalence was greater in areas with more natural area (RC = 0.35) and floral richness (RC = 0.23) but declined with greater floral density (RC = −0.21). Greater floral richness was also associated with increased DWV prevalence in *B. impatiens* (RC = 0.18) and *Lasioglossum* spp. (RC = 0.28). Higher landscape richness correlated with reduced DWV in *Lasioglossum* spp. (RC = −0.23), whereas greater natural area correlated with increased DWV in *E. pruinosa* (RC = 0.39).

Overall, although the specific links between habitat characteristics and viral prevalence differed among hosts and pathogens, the direction of the links was surprisingly consistent among different host species and viruses. A greater proportion of natural area and floral richness generally had amplifying effects on viral prevalence, regardless of the pollinator host species or virus. Therefore, habitat may have similar effects on different pathogens in different hosts.

### Are local‐ and landscape‐scale habitat characteristics indirectly associated with pathogen prevalence through habitat‐mediated changes in pollinator community diversity and/or abundance?

**FIGURE 4:**
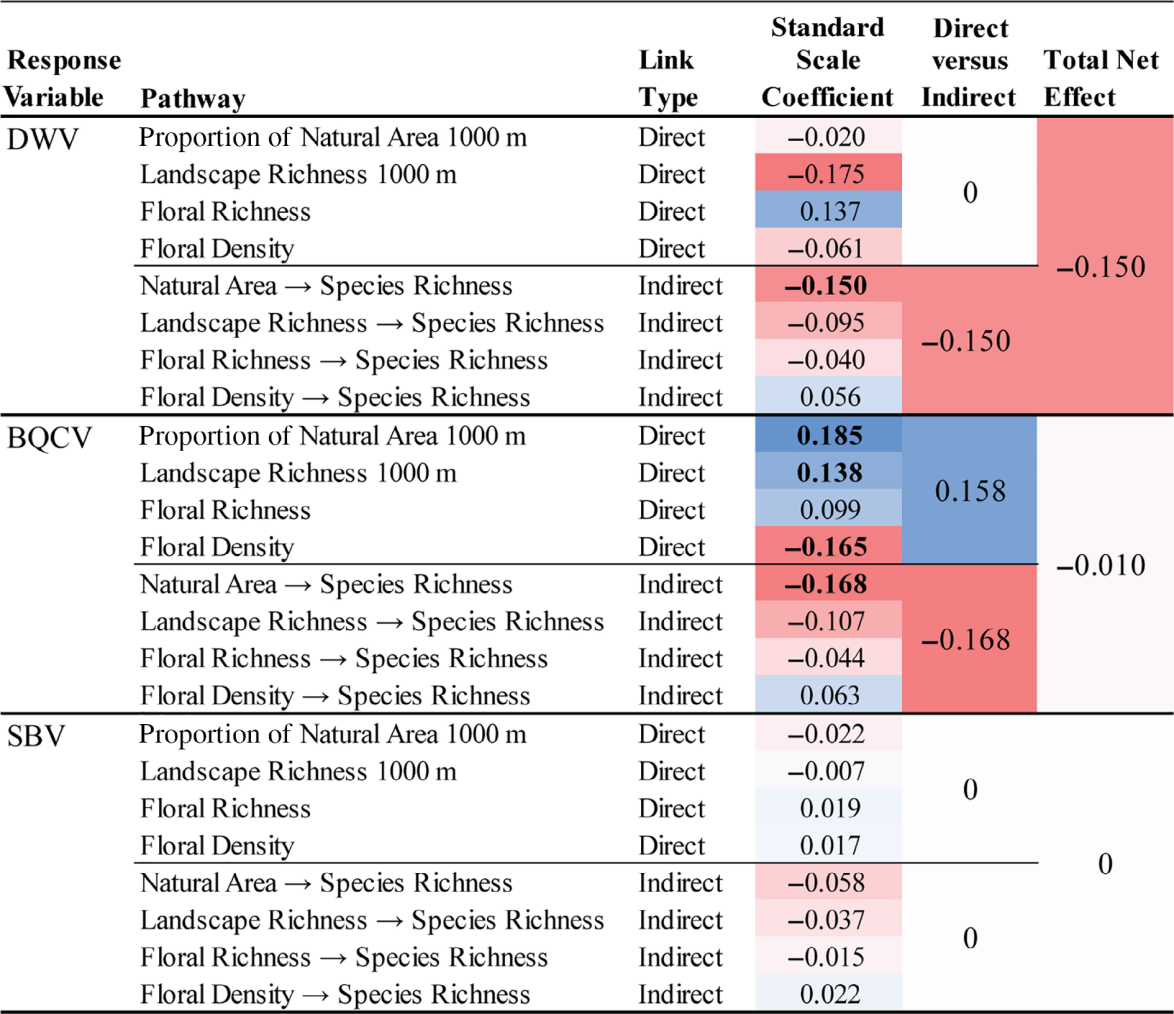
Comparison of scale‐standardized regression coefficients for direct and indirect pathways between habitat characteristics and deformed wing virus (DWV), black queen cell virus (BQCV), and sacbrood virus (SBV) prevalence. Indirect pathway coefficients are a product of the two significant direct pathway coefficients, from a habitat factor to species richness to viral prevalence. Then, for each virus, we compared the summed coefficients for all significant direct and indirect links, as well the total net effect of all significant pathways between habitat characteristics and viral prevalence. The indirect pathways through *Apis* and *Bombus* abundance are not shown because none of the paths were significant. The coefficients are colored based on the magnitude (darker = larger) and direction (red = negative; blue = positive) of the change in viral prevalence from a one‐SD change from the mean of each predictor. Standardized scale coefficients for both significant and nonsignificant pathways are included, with significant pathways from Figure 1b bolded. The nonsignificant pathway coefficients are not used in the calculations for the net effects (two right columns).

In addition to the direct effects of habitat on pathogen prevalence described earlier, habitat also indirectly influenced pathogen prevalence via habitat‐mediated effects on pollinator community richness. The overall path model including all species showed that greater proportion of natural area was strongly linked with increased pollinator species richness (RC = 0.91). Notably, greater species richness was linked with reduced DWV (RC = −0.14) and BQCV prevalence (RC = −0.16; Figures 1b and 2e). Therefore, the net indirect effect of increasing natural area among the study sites was to reduce DWV and BQCV prevalence by increasing pollinator species richness (Figure 4). A greater proportion of natural area was also linked with higher *A. mellifera* and *B. impatiens* abundance (RC = 0.83), but *A. mellifera* and *B. impatiens* abundance was not associated with differences in BQCV, DWV, or SBV prevalence (Figure 2f). Path models that included total pollinator abundance or species‐specific abundances similarly did not exhibit any links with viral prevalence (described further in Appendix S2: Section S3).

The species‐specific path models showed that habitat indirectly influenced pathogen prevalence via habitat‐mediated effects on pollinator community richness in *B. impatiens* and *Lasioglossum* spp., but not *A. mellifera* and *E. pruinosa* (Figure 3, Appendix S3: Tables S1–S4). Greater pollinator species richness reduced *B. impatiens* BQCV prevalence (RC = −0.21) and reduced *Lasioglossum* spp. DWV and BQCV prevalence (RC_DWV_ = −0.22; RC_BQCV_ = −0.54). Therefore, species richness influenced pathogen prevalence in some, but not all, host species. However, where biodiversity and pathogen prevalence are linked, pollinator species richness consistently had a negative impact on DWV and BQCV prevalence.

### Do direct habitat–disease and indirect biodiversity–disease pathways have similar relative strengths and directions?

In the overall model including all host species, the direct and indirect effects of habitat quality characteristics on DWV and BQCV prevalence had a similar relative strength. For each virus, the net effects of direct and indirect pathways were determined by summing the significant coefficients for each pathway type, and total net effects were calculated by summing all significant pathway coefficients. As habitat quality increased, the net effect of the direct habitat–disease pathways was generally positive in BQCV (SC = 0.158; Figure 4). Individual significant direct habitat–disease pathways varied considerably, with SCs ranging from −0.165 to 0.185. DWV had no significant direct habitat–disease pathways and SBV prevalence exhibited no significant direct or indirect links with habitat characteristics. In DWV and BQCV, greater habitat quality characteristics consistently had negative indirect effects mediated by pollinator species richness (indirect SC = DWV: −0.150; BQCV: −0.168; Figure 4). None of the indirect paths via combined *Apis* and *Bombus* abundance were significantly linked with viral prevalence. Overall, the total net effect of greater local‐ and landscape‐level habitat quality through all significant direct and indirect pathways strongly reduced DWV prevalence and had no net effect on BQCV and SBV prevalence (Net SC = DWV: −0.15; BQCV: −0.01; SBV: 0; Figure 4).

## DISCUSSION

Here we demonstrate that habitat simultaneously affects pathogen prevalence through direct habitat–disease relationships and by indirectly altering community diversity to produce a biodiversity–disease relationship. We investigated the influence of habitat quality characteristics on both pollinator communities and the prevalence of three viruses that have previously exhibited dilution effects. Habitat directly influences pathogen prevalence, perhaps through habitat effects on diet breadth, nutritional health, and susceptibility. Habitat also indirectly influences pathogen prevalence through its effect on pollinator communities. Thus far, most biodiversity–disease studies only examined the biodiversity–disease pathway, either as a direct link between host biodiversity and pathogen prevalence or as an indirect link where habitat characteristics are used as a proxy for biodiversity effects on pathogen prevalence. By simultaneously evaluating direct habitat–disease links alongside concurrent links among habitat, host biodiversity, and pathogen prevalence, we demonstrate that the pathways have comparable relative contributions to community‐wide pathogen prevalence.

### Habitat–disease relationships

We found clear evidence of a habitat–disease relationship that is separate from the link between biodiversity and viral prevalence, as multiple habitat characteristics at the local and landscape scales are directly associated with viral prevalence. These findings demonstrate that the proposed habitat–disease relationship could be an important, underexplored pathway contributing to variation in patterns of pathogen prevalence among communities and across spatial scales. In theory, habitat variables could be either positively or negatively correlated with pathogen prevalence. Here, our naïve expectation was that high‐quality habitat would be associated with reduced viral prevalence because of previous work illustrating that greater landscape richness, natural area, floral richness, and floral density are linked with improved pollinator nutrition and increased immune function (Alaux et al., 2010; Donkersley et al., 2014) and, therefore, are likely to facilitate greater resistance to infection. Instead, we observe both positive and negative direct links between habitat characteristics and viral prevalence. For example, floral density is linked with reduced BQCV prevalence, whereas a greater proportion of natural area and landscape richness are linked with increased BQCV prevalence. Host species‐specific viral prevalence tended to increase with greater habitat quality (e.g., more natural area, greater floral richness, higher landscape richness), though there was considerable variation in the topography of specific habitat–disease links among different viruses infecting different host species. Nutrition effects on infection outcomes in insects often vary among different hosts and pathogens, and more mechanistic studies are needed to explain these differences (Cotter & Al Shareefi, 2022). This variability in habitat–disease relationships suggests that different habitat metrics may have context‐dependent interactions with host health for different host species and pathogens through changes in host exposure, nutrition, susceptibility, and immune function.

Habitat may directly influence viral prevalence through multiple mechanisms, including effects on host nutrition and foraging patterns. Habitat characteristics that reduce viral prevalence may be closely associated with high‐quality diets that improve bee health. Bees with diverse or high‐protein pollen diets have better nutritional status, greater immune gene expression, and lower pathogen loads for multiple bee pathogens (DeGrandi‐Hoffman et al., 2010; Di Pasquale et al., 2013). Furthermore, some flowers contain phytochemicals that can confer medicinal benefits to bees by reducing pathogen loads or increasing immune gene expression (Palmer‐Young et al., 2017; Richardson et al., 2015). McNeil et al. (2020) found that bumblebees have reduced BQCV and DWV loads in areas with greater floral availability and greater immune gene expression when bees forage in areas with greater grassland cover. Therefore, diverse and abundant floral resources across scales may allow pollinators to forage on diverse or preferred flowers with high‐quality resources (e.g., protein‐rich pollen and/or medicines) (Gherman et al., 2014; Vaudo et al., 2016) to confer health benefits such as reduced susceptibility or better outcomes of infection (i.e., survival and/or lower pathogen loads).

Differences in habitat quality may influence bee foraging density or behaviors, thereby altering exposure to pathogens and parasites transmitted on flowers. This study and that of McNeil et al. (2020) both show that greater floral density correlated with reduced BQCV, especially in bumblebees. Local patches with greater floral abundance could reduce the effective foraging density of pollinators and result in reduced pathogen transmission. However, habitat effects at the landscape scale may alter bees' choice of which patches in the landscape to forage in, with potential consequences for pathogen transmission. We found that pollinators in areas with more land‐cover types (i.e., landscape richness) and natural area had greater BQCV prevalence. Figueroa et al. (2020) found a similar relationship where simpler landscapes with greater agricultural cover reduced parasite prevalence in the pollinator community by increasing the dietary breadth of dominant, generalist bumblebees. Therefore, viral transmission potential on flowers may be altered based on bee foraging choices in different habitats.

The positive link between high‐quality habitat and viral prevalence may also be due to increased pathogen proliferation and subsequent transmission in high‐quality environments where hosts can access better or additional resources that the pathogen can utilize. For example, some pathogens replicate more quickly in hosts with high‐quality nutrition by exploiting the extra resources harbored by the host (Penczykowski et al., 2014). Future studies are needed to test the mechanisms by which each habitat characteristic may mediate higher or lower pathogen prevalence by investigating host nutrition, behavior, immune function, exposure, and susceptibility to disease.

### Habitat–driven biodiversity–disease relationships

In addition to habitat directly influencing pathogen prevalence, habitat characteristics indirectly affect pathogen prevalence by changing pollinator community species richness. Importantly, habitat characteristics indirectly mediate biodiversity–disease relationships to produce dilution effects. Greater proportion of natural area at the landscape scale is positively linked with pollinator species richness, and greater species richness is correlated with reduced DWV and BQCV prevalence. Habitat‐driven changes in host community species richness could alter the rate of encounters with infected individuals on flowers (encounter reduction) or the rate of transmission through consumption of virus‐contaminated pollen (transmission reduction)—two of the key dilution effect mechanisms (Keesing et al., 2006). Figueroa et al. (2020) found that greater plant–pollinator network connectance led to a reduction in the variance of parasite prevalence among different species in the bee communities, diluting prevalence in highly infected species but increasing prevalence in less infected species. In our study, we found that more natural area surrounding our small agricultural fields increased pollinator species richness, potentially increasing the connectance of interacting bees within the field, which in turn led to reduced viral prevalence. Our results highlight the critical role that local‐ and landscape‐scale habitat characteristics may play in altering the outcomes of biodiversity–disease relationships.

It would be reasonable to expect that habitat quality should increase pollinator abundance, which should in turn increase transmission and therefore viral prevalence. We found that greater natural area strongly increased total pollinator species abundance and species‐specific abundances. However, contrary to our expectations, none of the pollinator abundance metrics were directly associated with community‐wide or host species‐specific viral prevalence. These results corroborate our previous findings that species richness is linked with viral prevalence, whereas species abundance is not (Fearon & Tibbetts, 2021). Notably, viral prevalence was not strongly affected by the abundance of highly competent host species, such as honey bees and bumblebees. These findings suggest that variation in viral prevalence among communities does not necessarily correspond to habitat‐mediated differences in pollinator population sizes or to the abundance of competent species that typically have high viral prevalence.

### Relative effects of habitat–disease and biodiversity–disease relationships

The relative magnitude of individual direct habitat–disease links and habitat‐mediated biodiversity–disease links were comparable, but the directionality of the pathways differed. Greater species richness was linked with lower viral prevalence, whereas greater habitat quality had mixed positive and negative associations with viral prevalence. Altogether, the net effect of increasing habitat quality through all significant direct and indirect links between habitat, biodiversity, and viral prevalence resulted in the greatest reduction in DWV prevalence and no net change in BQCV and SBV prevalence (Figure 4). This result corroborates previous evidence of dilution effects for DWV in multiple pollinator host species (Fearon & Tibbetts, 2021) but demonstrates that the effects of habitat–disease pathways may be sufficient to counteract biodiversity–disease pathways for BQCV prevalence. The difference in these observed patterns may be due to DWV and BQCV pathologies. BQCV is considered relatively benign compared to recently increasing DWV virulence in honey bees and bumblebees, though less is known about viral virulence in other wild bees (McMahon et al., 2016; Tehel et al., 2020). Our structural equation model provides a more detailed view of the underlying mechanisms that contribute to the patterns of pathogen prevalence. Therefore, including the effects of direct and indirect pathways between habitat and pathogen prevalence provides a clearer understanding of the sources of variation in pathogen prevalence among communities.

### Trade‐offs of various infection metrics

The specific infection metric used to estimate disease risk (e.g., pathogen prevalence, severity, or diversity) may influence habitat–disease and biodiversity–disease patterns (Roberts & Heesterbeek, 2018). Here, we used binary infection presence or absence, which is useful for understanding differences in patterns of prevalence among host species. However, other quantitative measures, such as viral titer or severity of infection, could provide complementary information about disease risk (Manley, Temperton, Boots, & Wilfert, 2019). For example, hosts with access to high‐quality resources may be able to launch an effective immune response to mitigate infection and keep viral titers low (DeGrandi‐Hoffman et al., 2010) or may tolerate infection better (i.e., reduced mortality) (Dolezal et al., 2019). We observed no strong correlations between habitat, pollinator community, and viral prevalence within honey bees (*A. mellifera*, Figure 3a), likely because of consistently high viral prevalence (Fearon & Tibbetts, 2021). However, there may be differences in honey bee viral titers that could influence transmission (Alger et al., 2019; Manley, Temperton, Doyle, et al., 2019). Furthermore, pathogen diversity metrics may demonstrate complex direct and indirect relationships with habitat and host biodiversity. A recent study found that increased habitat disturbance indirectly reduced intestinal parasite diversity by increasing small‐mammal species richness, but habitat disturbance did not directly affect parasite diversity in the focal, generalist host (Schwensow et al., 2022). These findings appear to contrast with our results, perhaps due to the choice of infection metric: parasite diversity instead of parasite prevalence. Greater pathogen diversity may lead to reduced pathogen prevalence in a focal host due to competition for hosts (Johnson et al., 2013), suggesting that choice of infection metric may produce qualitatively different patterns between habitat, host diversity, and disease risk.

### Potential for habitat–disease relationships in other host–pathogen systems

Habitat–disease relationships may explain some of the variability observed in biodiversity–disease relationships across host–pathogen systems. The literature shows inconsistent evidence for dilution effects, even when comparing the same host–pathogen system in different locations (Salkeld et al., 2013; Wood et al., 2014, 2017; Wood & Lafferty, 2013). Part of this variation may be explained by differences in habitat characteristics across sites via the habitat–disease relationship. For example, several hantaviruses have shown evidence of dilution effects (Dearing & Dizney, 2010; Khalil et al., 2016), but the landscape quality gradient used to establish biodiversity gradients can independently contribute to variable hantavirus prevalence (Dearing & Dizney, 2010; Langlois et al., 2001). Furthermore, poorer quality habitats reduce rodent immune function (Demas & Nelson, 1998) and decrease infected rodent winter survival (Kallio et al., 2007). These data suggest that habitat quality characteristics could have direct habitat–disease impacts on hantavirus prevalence via nutrition and/or immune function that are separate from previously studied biodiversity–disease and host density mechanisms.

## CONCLUSIONS

We show that habitat characteristics are both directly linked with viral prevalence and indirectly linked to viral prevalence through habitat effects on bee host species richness. Specifically, the direction of the individual habitat–disease pathways were quite variable among habitat characteristics, pathogens, and host species. Habitat‐driven increases in pollinator species richness were linked with reduced viral prevalence, whereas the habitat‐driven increases in pollinator abundance were not associated with any changes in viral prevalence. Habitat–disease and biodiversity–disease pathways had similar magnitudes, but their net directions varied. Overall, the combined net effect of greater habitat quality through all significant direct and indirect pathways reduced prevalence in one of the three viruses examined, whereas a second virus showed that the habitat and biodiversity pathways may counteract each other. These results indicate that habitat–disease relationships are important in mediating pathogen prevalence and could contribute to variability in apparent biodiversity–disease relationships observed in other host–pathogen systems. Habitat is an important driver in the complex interactions between hosts and pathogens since it both changes species interactions and alters host susceptibility and immunity.

## AUTHOR CONTRIBUTIONS

Michelle L. Fearon and Elizabeth A. Tibbetts developed manuscript ideas, Michelle L. Fearon conducted data collection and analyses with advice from Chelsea L. Wood and Elizabeth A. Tibbetts. Michelle L. Fearon wrote the first draft, and all authors contributed to revisions.

## CONFLICT OF INTEREST

The authors declare no conflict of interest.

## Supporting information


Appendix S1.

Appendix S2.

Appendix S3.

## Data Availability

All data and code (Fearon et al., 2022) used for the analyses and figures are available in Dryad at https://doi.org/10.5061/dryad.mkkwh710c. All sequences produced from this work are stored in GenBank (Accession Numbers: MN900314–MN900321 and MN902093–MN902138). Data sets utilized for this research are available for download by year at the following citation and link: USDA National Agricultural Statistics Service Cropland Data Layer (2015–2016). Published crop‐specific data layer [online] available at https://nassgeodata.gmu.edu/CropScape/ (accessed April 25, 2017; 2015 data verified February 12, 2016; 2016 data verified January 30, 2017), USDA‐NASS, Washington, DC.
