# Supplementary material for: Habitat quality influences pollinator pathogen prevalence through both habitat–disease and biodiversity–disease pathways
Source: Ecology. 2023 Jan 3;104(2):e3933. doi: 10.1002/ecy.3933 (PMC10078577; doi:10.1002/ecy.3933)
Supplement: Supplementary file 1 — Appendix S1. [file ECY-104-0-s002.pdf]

Appendix S1

Manuscript title: Habitat Quality Influences Pollinator Pathogen Prevalence Through Both Habitat–Disease and Biodiversity–Disease Pathways

Authors: Michelle L. Fearon, Chelsea L. Wood, Elizabeth A. Tibbetts

Journal: Ecology

Table S1: Field site abbreviation, farm name, sampling year, dates of each visit to field site (mm/dd/yyyy), and zone, easting and northing coordinates in the UTM GPS system. All field sites are in the Lower Peninsula of Michigan, USA.

Code	Farm Name	Year	First Visit	Second visit	Zone	Easting	Northing
W	Wasem Fruit Farm	2015	7/22/2015	7/30/2015	17T	282985	4668596
G	Gust Brother's Pumpkin Farm	2015	7/26/2015	8/5/2015	17T	267978	4625139
BC	The Blast Corn Maze (Nixon Farms)	2015	7/24/2015	8/2/2015	17T	264841	4692908
BJ	BJ Farm	2015	7/23/2015	7/31/2015	17T	281032	4630188
S	Stone Coop Farm	2015	8/7/2015	8/21/2015	17T	271906	4704446
BP	Brimley's Pumpkin Patch	2016	8/10/2016	8/26/2016	16T	714474	4716740
T	Tantré Farm	2016	8/18/2016	9/2/2016	16T	738575	4681735
K	Kapnick Orchards	2016	8/21/2016	8/28/2016	17T	257729	4648607
SP	Spicer Orchards	2016	8/14/2016	9/1/2018	17T	274397	4729038
PR	Peacock Road Farms	2016	7/26/2016	8/23/2016	16T	714244	4746884
GT	Green Things Farm	2016	8/17/2016	8/24/2016	17T	276741	4689607
E	Erwin Orchards	2016	7/27/2016	8/22/2016	17T	280997	4708908
PL	Plymouth Orchards	2016	8/11/2016	8/30/2016	17T	289557	4690343

Table S2. All observed landcover categories within 1000 m of the 13 field sites from the USDA Crop Land Data Layer. Each landcover type was categorized as 'Agriculture', 'Developed', or 'Natural' using the National Land Cover Database legend https://www.mrlc.gov/data/legends/national-land-cover-database-2019-nlcd2019-legend. 'Natural' landcover types were used to calculate the proportion of natural area, and all types of landcover present were used to calculate the landscape richness within 1000 m.

Category	Sub	Type
Agriculture	Alfalfa	Alfalfa
Agriculture	Grain	Barley
Agriculture	Corn	Corn
Agriculture	Other	Fallow Idle Cropland
Agriculture	Grain	Oats
Agriculture	Hay	Other Hay Non-Alfalfa
Agriculture	Corn	Pop Corn
Agriculture	Grain	Rye
Agriculture	Grass	Sod or Grass Seed
Agriculture	Corn	Sorghum
Agriculture	Bean	Soybeans
Agriculture	Grain	Speltz
Agriculture	Flowers	Sunflowers
Agriculture	Corn	Sweet Corn
Agriculture	Grain	Triticale
Agriculture	Grain	Winter Wheat
Agriculture	Orchard	Apples
Agriculture	Christmas Trees	Christmas Trees
Agriculture	Vegetables	Cabbage
Agriculture	Cucurbits	Cucumbers
Agriculture	Bean	Dry Beans
Agriculture	Herbs	Herbs
Agriculture	Other	Misc. Vegetables and Fruits
Agriculture	Root Veg	Onions
Agriculture	Solanums	Peppers
Agriculture	Root Veg	Potatoes
Agriculture	Cucurbits	Pumpkins
Agriculture	Root Veg	Sugar beets
Agriculture	Solanums	Tomatoes
Developed	High	Developed High Intensity
Developed	Low	Developed Low Intensity
Developed	Med	Developed Medium Intensity
Developed	Open	Barren
Developed	Open	Developed OpenSpace
Natural	Forest	Deciduous Forest
Natural	Forest	Evergreen Forest
Natural	Forest	Mixed Forest
Natural	Range	Clover Wildflowers
Natural	Range	Grass Pasture
Natural	Range	Shrubland
Natural	Wetland	Herbaceous Wetlands
Natural	Wetland	Woody Wetlands

Table S3: The number of individuals tested for the presence of DWV, BQCV, and SBV. Where less than 20 individuals for a species were collected, then all collected individuals for that species were tested for viral prevalence.

Year	***2015***					***2016***								
Species Names	**W**	**BJ**	**BC**	**G**	**S**	**PR**	**E**	**BP**	**PL**	**SP**	**GT**	**T**	**K**	**Species totals**
Apis mellifera	20	16	21	20	20	20	20	20	20	20	20	8	9	**234**
Bombus impatiens	13	30	13	3	30	19	20	20	20	20	20	20	20	**248**
Lasioglossum spp.	12	19	22	2	19	20	19	20	20	20	20	20	20	**233**
Eucera pruinosa	21	16	20	3	20	20	20	6	20	6	20	1	0	**173**

Table S4: Primer pair sequences for positive and negative strand BQCV, DWV, and SBV screens and 18S rRNA gene, RT-PCR annealing temperatures, PCR product size, and reference source for the design of each primer set.

**Virus/ Gene**	**Purpose**	**Primer name**	**Primer sequence**	**T_a**	**Product size**	**Ref**
**BQCV**	RT-PCR screen	BQCV 3'UTR F	TGGTCAGCTCCCACTACCTTAAAC	57 ºC	700bp	(*1*)
		BQCV 3'UTR R	GCAACAAGAAGAAACGTAAACCAC			
**DWV**	RT-PCR screen	DWV VP1a F	CTCGTCATTTTGTCCCGACT	56 ºC	424bp	(*2*)
		DWV VP1a R	TGCAAAGATGCTGTCAAACC			
**SBV**	RT-PCR screen	SBV VP1b F	GCACGTTTAATTGGGGATCA	55 ºC	693bp	(*2*)
		SBV VP1b R	CAGGTTGTCCCTTACCTCCA			
**18 S rRNA**	RT-PCR screen (bee positive control)	18S H17F	AAATTACCCACTCCCGGCA	58 ºC	784bp	(*3*)
		18S H35R	TGGTGAGGTTTCCCGTGTT			
**DWV**	negative strand specific cDNA synthesis	Tag-F15	agcctgcgcaccgtggTCCATCAGGTTCTCC AATAACGGA	na	na	(*4*)
**DWV**	negative strand specific RT-PCR	B23	CCACCCAAATGCTAACTCTAAGCG	54.5 ºC	450bp	(*4*)
**BQCV**	negative strand specific cDNA synthesis	Tag-BQCV-sense	agcctgcgcaccgtggTCAGGTCGGAATAA TCTCGA	na	na	(*5*)
**general**	negative strand specific RT-PCR	Tag	AGCCTGCGCACCGTGG	na	na	(*5*)
**BQCV**	negative strand specific RT-PCR	BQCV-antisense	GCAACAAGAAGAAACGTAAACCAC	55 ºC	420bp (with tag)	(*5*)
**SBV**	negative strand specific cDNA synthesis	Tag-SB7f	agcctgcgcaccgtggGGAGATGTTAGAAA TACCAACCGATTCC	na	na	(*6*)
**SBV**	negative strand specific RT-PCR	SB8R	CCATTAAACAAATCGGTATAAGAG TCCACT	57 ºC	200bp	(*6*)

References

1. M. Benjeddou, N. Leat, M. Allsopp, S. Davison, Detection of Acute Bee Paralysis Virus and Black Queen Cell Virus from Honeybees by Reverse Transcriptase PCR. *Appl. Environ. Microbiol.* **67**, 2384–2387 (2001).
2. R. Singh, A. L. Levitt, E. G. Rajotte, E. C. Holmes, N. Ostiguy, D. Vanengelsdorp, W. I. Lipkin, C. W. Depamphilis, A. L. Toth, D. L. Cox-Foster, RNA viruses in hymenopteran pollinators: evidence of inter-taxa virus transmission via pollen and potential impact on non-Apis hymenopteran species. *PLoS One*. **5**, e14357 (2010).
3. S. Cardinal, J. Straka, B. N. Danforth, Comprehenisve phylogeny of apid bees reveals the evolutionary origins and antiquity of cleptparasitism. *Proc. Natl. Acad. Sci.* **107**, 16207–16211 (2010).
4. C. Yue, E. Genersch, RT-PCR analysis of Deformed wing virus in honeybees (Apis mellifera) and mites (Varroa destructor). *J. Gen. Virol.* **86**, 3419–3424 (2005).
5. W. Peng, J. Li, H. Boncristiani, J. P. Strange, M. Hamilton, Y. Chen, Host range expansion of honey bee Black Queen Cell Virus in the bumble bee, Bombus huntii. *Apidologie*. **42**, 650–658 (2011).
6. H. R. Gong, X. X. Chen, Y. P. Chen, F. L. Hu, J. L. Zhang, Z. G. Lin, J. W. Yu, H. Q. Zheng, Evidence of *Apis cerana* Sacbrood virus infection in *Apis mellifera*. *Appl. Environ. Microbiol.* **82**, 2256–2262 (2016).
