# Supplementary material for: Habitat quality influences pollinator pathogen prevalence through both habitat–disease and biodiversity–disease pathways
Source: Ecology. 2023 Jan 3;104(2):e3933. doi: 10.1002/ecy.3933 (PMC10078577; doi:10.1002/ecy.3933)
Supplement: Supplementary file 2 — Appendix S2. [file ECY-104-0-s001.pdf]

Appendix S2

Manuscript title: Habitat Quality Influences Pollinator Pathogen Prevalence Through Both Habitat–Disease and Biodiversity–Disease Pathways

Authors: Michelle L. Fearon, Chelsea L. Wood, Elizabeth A. Tibbetts

Journal: Ecology

Section S1: Additional details about the main path model

All analyses were performed in R version 4.1.1 (R Core Team 2021) and models were fit and evaluated with the *piecewiseSEM* package (Lefcheck 2016). All habitat and pollinator community variables were scaled and centered, pollinator species richness and *E. pruinosa* abundance were square root transformed, and proportion of natural area was arcsine square root transformed (details in **Appendix S2: Table S1**). All other species-specific abundances were normally distributed without transformation. The main path model is presented in **Figure 1b** and **Appendix S2: Table S3** and included combined *A. mellifera* and *B. impatiens* abundance (hereafter called the 'main path model').

The models also included correlated errors between pollinator species richness and abundance to control for the positive correlation between these factors, and among the three viruses since presence of one virus in a host could impact the likelihood of infection with another virus.

We evaluated all component models for their model assumptions (*olsrr* package, Hebbali 2020). All main factors in the models had Variance Inflation Factors <4.5 and Pearson's correlations <0.75, indicating that there were acceptable levels of collinearity among factors in the model (Dormann et al. 2013; **Appendix S2: Table S4**). None of the component GLMMs were overdispersed. None of the component models showed evidence of spatial autocorrelation in model residuals (Moran's I test, *ape* and *DHARMa* packages; **Appendix S2: Table S5**) (Paradis and Schliep 2018, Hartig 2020).

The viral prevalence GLMMs include the prevalence of all four host species combined, with host species and visit to each site nested within site as random effects. In some models, the nested random effects of visit within site were singular, indicating that the random effect did not explain any variation in the model. However, we left all the random effects in the model to account for bees that were collected at the same time and from the same sites that may have more similar viral prevalence to each other. These models will capture overall patterns of habitat and pollinator community effects on BQCV, DWV, and SBV prevalence within the communities, while also accounting differences in viral prevalence based on host species, as we previously found in Fearon and Tibbetts 2021.

Since the main path model was completely saturated (included all possible links), we could not assess the goodness of fit (χ^2=0, p=1) nor conduct tests of directed separation for the full model. To better assess the goodness of fit for the model, we used model simplification where we

removed select paths that had little support in the model with p-values greater than 0.8, following (Grace 2020), to produce a similar model to the main model that was no longer fully saturated. The χ^2 test of goodness of fit determines whether the hypothesized relationships in the model deviate substantially from the relationships found in the data. Therefore, a high p-value for the χ^2 statistic indicates that the data fits the model-implied relationships (H_0), while a low p-value suggests that a different model structure would be better (H_1). The tests of directed separation evaluate whether there are any significant paths that are missing from the model, where missing paths with a significant path coefficient would produce a low p-value for the Fisher's C statistic. In the main model, two paths were temporarily removed (proportion of natural area on DWV prevalence, p=0.858, and landscape richness on SBV prevalence, p=0.912), which produced Chi-squared and direction of separation test statistics that indicated that the data fits the model well with p-values well above the 0.05 threshold (χ^2=0.044, p=0.978; Fisher's C=0.489; p=0.975). Furthermore, the topography of significant paths and their relative magnitudes did not change in the simplified model. All paths are included in the main path model that is presented in the main text.

Section S2: Parallel path model with estimated species richness
Though our main analyses utilize observed pollinator species richness, we also conducted a parallel analysis with estimated species richness because it is rare to reach an asymptote when sampling invertebrate communities (Novotný and Basset 2000, Gotelli and Colwell 2001). We generated individual-based rarefaction curves (*iNext* package, Hsieh et al. 2016) and determined that estimated species richness for the average number of pollinators captured at each site (mean = 338 across all 13 field sites; rarefaction curves previously published in appendices of Fearon and Tibbetts 2021). This estimates species richness at a consistent number of individuals sampled for each site. Then we ran a parallel path model as described in the main text with estimated species richness instead of observed species richness.

The estimated species richness path model was completely saturated; therefore, we could not test model fit with d-separation or Chi-squared tests. However, we conducted a similar temporary model simplification procedure as described above for the main model and removed selected paths that had p-values greater than 0.7 to assess goodness of fit. Here we used a lower p-value threshold for removing paths because there were not any paths that had a p-value over 0.8. Three paths were removed (proportion of natural area on DWV prevalence, p= 0.729, landscape richness and floral richness on SBV prevalence, p=0.715 and p=747, respectively), which produced test statistics that indicated good model fit with the data (χ^2=0.373, p=0.946; Fisher's C=1.984; p=0.921). We did not remove the total density on *Apis* and *Bombus* abundance pathway even though the p-value was > 0.7 because the d-separation test indicated that path needed to be included in the model.

We found that the topography of significant pathways in the estimated richness path model varied slightly from main path model presented with the lowest AIC but was overall consistent with the results from the other versions of the main path model described above. Additionally, the estimated richness path model had a ΔAIC of 9.11 higher than the model with the lowest AIC, therefore our results were robust to our choice of species richness measurement (**Appendix S2: Table S11**).

Section S3: Methods and results for path models with total pollinator abundance or species-specific host abundance instead of combined *A. mellifera* and *B. impatiens* abundance

Overall, path models that included total pollinator abundance or each of the species-specific host abundance had relatively similar topography of significant pathways and directionality to the main path model presented in **Figure 1b**. The model that contained *B. impatiens* abundance was the second-best model based on lowest AIC, followed by the models including total pollinator abundance, *E. pruinosa* abundance, *Lasioglossum* spp. abundance, and *A. mellifera* abundance (**Appendix S2: Table S2**). Most importantly, none of the pollinator abundance terms in any of these models were significantly linked with BQCV, DWV, nor SBV prevalence.

Here, we will mainly focus on the key differences among these models in significant pathways from the main path model presented in **Figure 1b** that included combined *A. mellifera* and *B. impatiens* abundance (hereafter called the 'main path model') and refer to the corresponding model output table with additional details for pathway coefficients, p-values, and R^2. In general, all differences among these models reflect changes in whether a subset of pathways are significant in one model versus another, but none of the significant pathways change in directionality (i.e., a significant positive link does not become a significant negative link in another model). In most cases where a link changes from significant to non-significant (or vice versa), the p-values for the statistically insignificant paths remain < 0.1.

The model that includes *B. impatiens* abundance has the same topography of significant pathways as the main path model (**Appendix S2: Table S8**). In the total pollinator abundance path model, only major difference from the main path model is that the negative link between pollinator species richness and DWV prevalence is no longer significant (RCF = –0.121, p = 0.077; **Appendix S2: Table S6**). The *A. mellifera* abundance path model does not include the link between proportion of natural area and *A. mellifera* abundance (RCF = 0.341, p = 0.5), nor the link between pollinator species richness and DWV prevalence (RCF = –0.071, p = 0.36). But it does now include a significant positive pathway between floral richness and BQCV prevalence (RCF = –0.086, p = 0.046; **Appendix S2: Table S7**). Similarly, in the *Lasioglossum* spp. abundance path model, proportion of natural area is not significantly linked with *Lasioglossum* spp. abundance (RCF = 0.81, p = 0.052) and pollinator species richness is not correlated with DWV prevalence (RCF = –0.134, p = 0.072; **Appendix S2: Table S9**). Finally, in the E. pruinosa abundance path model, proportion of natural area is not significantly linked with *E. pruinosa* abundance (RCF = –0.244, p = 0.56) and pollinator species richness is not correlated with DWV prevalence (RCF = –0.121, p = 0.059). However, floral richness is significantly positively associated with greater BQCV prevalence (RCF = 0.065, p = 0.042; **Appendix S2: Table S10**).

Table S1: Description of variables included in the path models. Table contains the name, type of variable, applied transformations, mean and standard deviation of untransformed values, and a brief description of each variable. DWV, BQCV, and SBV prevalence includes all four host species in the main model, and only a single host species prevalence in the species-specific models. All variables except for viral prevalence were scaled and centered.

Variable Name	Type	Transformation*	Mean	SD	Description
**DWV**	binary	none	na	na	Presence (1) or absence (0) of DWV in individual bees.
**BQCV**	binary	none	na	na	Presence (1) or absence (0) of BQCV in individual bees.
**SBV**	binary	none	na	na	Presence (1) or absence (0) of SBV in individual bees.
**Species Richness**	integer	square root	28.46	13.28	The number of pollinator host species detected per site.
**Total Abundance**	integer	none	333.08	163.86	The total number of pollinator individuals detected per site.
***Apis mellifera* Abundance**	integer	none	38.85	20.84	The number of *Apis mellifera* individuals detected per site.
***Bombus impatiens* Abundance**	integer	none	80.38	53.34	The number of *Bombus impatiens* individuals detected per site.
***Apis* and *Bombus* Abundance**	integer	none	119.23	63.17	The number of *Apis mellifera* and *Bombus impatiens* individuals detected per site.
***Lasioglossum* spp. Abundance**	integer	none	93.69	93.55	The number of *Lasioglossum* spp. individuals detected per site.
***Eucera pruinosa* Abundance**	integer	square root	42.85	52.62	The number of *Eucera pruinosa* individuals detected per site.
**Landscape Richness**	integer	none	20.15	3.46	The number of different landcover types that occur within a 1000m radius of each site.
**Natural Area**	continuous	arcsine square root	0.52	0.20	The proportion of area within a 1000m that was classified as 'Natural Area' for each site. We classified forest, wetland, meadow landcover types as 'Natural Area'. Continuous between 0 and 1.
**Floral Richness**	integer	none	10.69	4.80	The number of different floral species detected at each site.
**Floral Density (m^2)**	continuous	none	32.42	23.63	The density of all flowers per m^2 detected at each site.

Table S2: Comparison of main path model AIC and ΔAIC. All path models have the same topography and model structure, but the pollinator abundance factor varies among the models to include either Total pollinator abundance, *Apis mellifera* abundance, *Bombus impatiens* abundance, combined *Apis* and *Bombus* abundance, *Lasioglossum* spp. abundance, and *Eucera pruinosa* abundance. Different path models are indicated by the type of pollinator abundance included in the model.

**Path Model**	**AIC**	**ΔAIC**
Apis + *Bombus* abundance	2506.9	0
Bombus impatiens abundance	2507.3	0.37
Total pollinator abundance	2512.4	5.48
Eucera pruinosa abundance	2514.4	7.47
Lasioglossum spp.	2517.0	10.14
Apis mellifera abundance	2519.7	12.78

Table S3: The full path model statistics for main model shown in **Figure 1B** that had the lowest AIC and included the combined *Apis mellifera* and *Bombus impatiens* abundance. The table shows the unstandardized estimated pathway coefficients, range-standardized estimated coefficients, scale standardized estimated coefficients, standard error, degrees of freedom, critical value, and p-value for each path. R^2 is shown for each response variable. The last four rows are correlated errors. Significant pathway coefficients and p-values are bolded, with negative coefficients in red and positive coefficients in black.

Response variable	Predictor variable	Estimate	Range Std Estimate	Scale Std Estimate	Std Error	DF	Critical Value	P-value		R^2
Species Richness	Prop. Natural Area 1000m	**0.633**	**0.910**	**0.633**	0.199	8	3.173	**0.013**	*	0.70
	Landscape Richness 1000m	0.403	0.438	0.403	0.241	8	1.673	0.133		
	Floral Richness	0.167	0.179	0.167	0.256	8	0.653	0.532		
	Floral Density	-0.236	-0.209	-0.236	0.278	8	-0.851	0.419		
Apis + *Bombus* Abundance	Prop. Natural Area 1000m	**0.567**	**0.834**	**0.567**	0.186	8	3.049	**0.016**	*	0.71
	Landscape Richness 1000m	0.439	0.488	0.439	0.225	8	1.954	0.086		
	Floral Richness	-0.094	-0.103	-0.094	0.239	8	-0.395	0.703		
	Floral Density	0.084	0.076	0.084	0.259	8	0.323	0.755		
DWV	Prop. Natural Area 1000m	-0.042	-0.017	-0.020	0.234	888	-0.179	0.858		0.22
	Landscape Richness 1000m	-0.371	-0.112	-0.175	0.202	888	-1.837	0.066		
	Floral Richness	0.289	0.086	0.137	0.170	888	1.701	0.089		
	Floral Density	-0.130	-0.032	-0.061	0.188	888	-0.690	0.490		
	Species Richness	**-0.500**	**-0.138**	**-0.236**	0.231	888	-2.165	**0.030**	*	
	Apis + *Bombus* Abundance	0.352	0.095	0.167	0.246	888	1.432	0.152		
BQCV	Prop. Natural Area 1000m	**0.467**	**0.156**	**0.185**	0.187	888	2.497	**0.013**	*	0.41
	Landscape Richness 1000m	**0.348**	**0.088**	**0.138**	0.156	888	2.230	**0.026**	*	
	Floral Richness	0.251	0.062	0.099	0.131	888	1.916	0.055		
	Floral Density	**-0.416**	**-0.085**	**-0.165**	0.143	888	-2.904	**0.004**	**	
	Species Richness	**-0.670**	**-0.155**	**-0.266**	0.187	888	-3.587	**<0.001**	***	
	Apis + *Bombus* Abundance	-0.087	-0.020	-0.034	0.185	888	-0.470	0.638		
SBV	Prop. Natural Area 1000m	-0.058	-0.018	-0.022	0.184	888	-0.314	0.753		0.38
	Landscape Richness 1000m	-0.019	-0.005	-0.007	0.175	888	-0.110	0.912		
	Floral Richness	0.051	0.012	0.019	0.135	888	0.376	0.707		
	Floral Density	0.045	0.009	0.017	0.148	888	0.304	0.761		
	Species Richness	-0.246	-0.054	-0.092	0.189	888	-1.302	0.193		
	Apis + *Bombus* Abundance	0.163	0.035	0.061	0.205	888	0.796	0.426		
DWV	BQCV	**0.199**	**0.199**	**0.199**	NA	888	6.048	**<0.001**	***	
BQCV	SBV	**0.165**	**0.165**	**0.165**	NA	888	4.965	**<0.001**	***	
SBV	DWV	**0.180**	**0.180**	**0.180**	NA	888	5.446	**<0.001**	***	
Apis + *Bombus* Abundance	Species Richness	0.149	0.149	0.149	NA	13	0.475	0.323		

Table S4: Pearson's correlation between each continuous variable included in the main path model below the diagonal, with the corresponding p-values above the diagonal. Variables have the same transformations used in the path model. Significant coefficients and p-values are bolded, with negative correlations colored red and positive correlations colored black.

	DWV	BQCV	SBV	Species Richness	Total Abundance	*A. mellifera* Abundance	*B. impatiens* Abundance	*Apis + Bombus* Abundance	*Lasioglossum* Abundance	*E. pruinosa* Abundance	Landscape Richness	Natural Area	Floral Richness	Floral Density
**DWV**	--	**<0.001**	**<0.001**	**<0.001**	**0.001**	0.556	**<0.001**	**0.002**	**0.024**	0.605	**<0.001**	**0.001**	0.616	0.184
**BQCV**	**0.365**	--	**<0.001**	**0.003**	**0.005**	0.771	**0.028**	0.082	0.311	0.315	0.668	**0.03**	0.537	**0.02**
**SBV**	**0.266**	**0.312**	--	0.163	0.073	0.283	0.764	0.545	0.135	0.112	0.975	0.253	0.768	0.754
**Species Richness**	**-0.181**	**-0.101**	-0.047	--	**<0.001**	0.228	**<0.001**	**<0.001**	**<0.001**	**<0.001**	**<0.001**	**<0.001**	**<0.001**	**<0.001**
**Abundance**	**-0.116**	**-0.095**	-0.06	**0.517**	--	**<0.001**	**<0.001**	**<0.001**	**<0.001**	**<0.001**	**<0.001**	**<0.001**	0.33	**<0.001**
***A. mellifera* Abundance**	0.02	0.01	-0.036	0.041	**0.685**	--	**<0.001**	**<0.001**	**<0.001**	**<0.001**	**<0.001**	**<0.001**	**<0.001**	**<0.001**
***B. impatiens* Abundance**	**-0.13**	**-0.074**	-0.01	**0.815**	**0.726**	**0.348**	--	**<0.001**	**<0.001**	**<0.001**	**<0.001**	**<0.001**	**<0.001**	**<0.001**
***Apis + Bombus* Abundance**	**-0.102**	-0.058	-0.02	**0.695**	**0.833**	**0.621**	**0.951**	--	**<0.001**	**<0.001**	**<0.001**	**<0.001**	**<0.001**	**<0.001**
***Lasioglossum* Abundance**	**-0.076**	-0.034	-0.05	**0.117**	**0.84**	**0.703**	**0.408**	**0.573**	--	0.847	0.629	**<0.001**	**<0.001**	0.882
***E. pruinosa* Abundance**	0.017	-0.034	-0.053	**-0.224**	**0.155**	**0.255**	**-0.256**	**-0.13**	0.006	--	**<0.001**	**<0.001**	**<0.001**	**<0.001**
**Landscape Richness**	**-0.161**	-0.014	-0.001	**0.502**	**0.203**	**0.326**	**0.524**	**0.546**	0.016	**-0.222**	--	**<0.001**	**<0.001**	**<0.001**
**Natural Area**	**-0.112**	**-0.073**	-0.038	**0.677**	**0.7**	**0.374**	**0.694**	**0.704**	**0.512**	**-0.131**	**0.182**	--	**<0.001**	**<0.001**
**Floral Richness**	0.017	-0.021	0.01	**0.33**	0.033	**0.161**	**0.24**	**0.253**	**-0.258**	**0.141**	**0.333**	**0.253**	--	**<0.001**
**Floral Density**	-0.045	**-0.078**	0.011	**0.2**	**0.329**	**0.41**	**0.315**	**0.398**	0.005	**0.434**	**0.455**	**0.255**	**0.526**	--

Table S5: Moran's I spatial autocorrelation model output for each of the component models in the main path model.

Model Response Variable	Observed	Expected	SD	P-value
Species Richness	-0.140	-0.083	0.060	0.35
Abundance	-0.079	-0.083	0.060	0.95
A. mellifera Abundance	-0.075	-0.083	0.060	0.89
B. impatiens Abundance	-0.088	-0.083	0.059	0.94
A. mellifera and B. impatiens Abundance	-0.086	-0.083	0.058	0.97
Lasioglossum spp. Abundance	-0.083	-0.083	0.060	0.996
E. pruinosa Abundance	-0.068	-0.083	0.060	0.79
DWV	-0.119	-0.083	0.060	0.56
BQCV	-0.154	-0.083	0.059	0.23
SBV	-0.037	-0.083	0.060	0.44

Table S6: The full path model statistics for the path model that included the total pollinator abundance. The table shows the unstandardized estimated pathway coefficients, range-standardized estimated coefficients, standard error, degrees of freedom, critical value, and p-value for each path. R^2 is shown for each response variable. The last four rows are correlated errors. Significant pathway coefficients and p-values are bolded, with negative coefficients in red and positive coefficients in black.

Response variable	Predictor variable	Estimate	Range Std Estimate	Std Error	DF	Critical Value	P-value		R^2
Species Richness	Prop. Natural Area 1000m	**0.633**	**0.910**	0.199	8	3.173	**0.013**	*	0.70
	Landscape Richness 1000m	0.403	0.438	0.241	8	1.673	0.133		
	Floral Richness	0.167	0.179	0.256	8	0.653	0.532		
	Floral Density	-0.236	-0.209	0.278	8	-0.851	0.419		
Total Pollinator Abundance	Prop. Natural Area 1000m	**0.682**	**0.741**	0.210	8	3.255	**0.012**	*	0.65
	Landscape Richness 1000m	0.021	0.017	0.253	8	0.084	0.935		
	Floral Richness	-0.282	-0.228	0.269	8	-1.047	0.326		
	Floral Density	0.305	0.203	0.292	8	1.044	0.327		
DWV	Prop. Natural Area 1000m	0.140	0.056	0.258	888	0.540	0.589		0.22
	Landscape Richness 1000m	-0.240	-0.072	0.195	888	-1.229	0.219		
	Floral Richness	0.238	0.071	0.197	888	1.207	0.227		
	Floral Density	-0.082	-0.020	0.215	888	-0.382	0.703		
	Species Richness	-0.438	-0.121	0.248	888	-1.769	0.077		
	Total Pollinator Abundance	-0.016	-0.006	0.236	888	-0.069	0.945		
BQCV	Prop. Natural Area 1000m	**0.476**	**0.159**	0.185	888	2.566	**0.010**	*	0.41
	Landscape Richness 1000m	**0.313**	**0.079**	0.141	888	2.220	**0.026**	*	
	Floral Richness	0.229	0.057	0.138	888	1.664	0.096		
	Floral Density	**-0.393**	**-0.080**	0.151	888	-2.609	**0.009**	**	
	Species Richness	**-0.663**	**-0.154**	0.185	888	-3.596	**<0.001**	***	
	Total Pollinator Abundance	-0.100	-0.031	0.161	888	-0.618	0.536		
SBV	Prop. Natural Area 1000m	0.032	0.010	0.181	888	0.177	0.860		0.38
	Landscape Richness 1000m	0.047	0.011	0.148	888	0.318	0.750		
	Floral Richness	0.029	0.007	0.139	888	0.206	0.837		
	Floral Density	0.071	0.014	0.151	888	0.468	0.640		
	Species Richness	-0.213	-0.047	0.182	888	-1.174	0.240		
	Total Pollinator Abundance	-0.032	-0.009	0.174	888	-0.187	0.852		
DWV	BQCV	**0.199**	**0.199**	NA	888	6.030	**<0.001**	***	
BQCV	SBV	**0.163**	**0.163**	NA	888	4.910	**<0.001**	***	
SBV	DWV	**0.181**	**0.181**	NA	888	5.472	**<0.001**	***	
Total Pollinator Abundance	Species Richness	0.095	0.095	NA	13	0.301	0.385		

Table S7: The full path model statistics for the path model that included *Apis mellifera* abundance. The table shows the unstandardized estimated pathway coefficients, range-standardized estimated coefficients, standard error, degrees of freedom, critical value, and p-value for each path. R^2 is shown for each response variable. The last four rows are correlated errors. Significant pathway coefficients and p-values are bolded, with negative coefficients in red and positive coefficients in black.

Response variable	Predictor variable	Estimate	Range Std Estimate	Std Error	DF	Critical Value	P-value		R^2
Species Richness	Prop. Natural Area 1000m	**0.633**	**0.910**	0.199	8	3.173	**0.013**	*	0.70
	Landscape Richness 1000m	0.403	0.438	0.241	8	1.673	0.133		
	Floral Richness	0.167	0.179	0.256	8	0.653	0.532		
	Floral Density	-0.236	-0.209	0.278	8	-0.851	0.419		
Apis mellifera Abundance	Prop. Natural Area 1000m	0.213	0.341	0.302	8	0.705	0.501		0.25
	Landscape Richness 1000m	0.238	0.289	0.364	8	0.654	0.532		
	Floral Richness	-0.172	-0.206	0.387	8	-0.445	0.668		
	Floral Density	0.311	0.305	0.421	8	0.738	0.481		
DWV	Prop. Natural Area 1000m	-0.044	-0.018	0.253	888	-0.176	0.860		0.22
	Landscape Richness 1000m	-0.366	-0.110	0.213	888	-1.720	0.085		
	Floral Richness	0.255	0.076	0.172	888	1.487	0.137		
	Floral Density	-0.115	-0.028	0.191	888	-0.601	0.548		
	Species Richness	-0.255	-0.071	0.280	888	-0.911	0.362		
	Apis mellifera Abundance	0.228	0.057	0.192	888	1.189	0.234		
BQCV	Prop. Natural Area 1000m	**0.471**	**0.157**	0.196	888	2.399	**0.016**	*	0.41
	Landscape Richness 1000m	**0.349**	**0.088**	0.161	888	2.164	**0.030**	*	
	Floral Richness	**0.259**	**0.064**	0.130	888	1.992	**0.046**	*	
	Floral Density	**-0.420**	**-0.086**	0.143	888	-2.940	**0.003**	**	
	Species Richness	**-0.732**	**-0.170**	0.210	888	-3.483	**<0.001**	***	
	Apis mellifera Abundance	-0.058	-0.012	0.141	888	-0.411	0.681		
SBV	Prop. Natural Area 1000m	0.118	0.037	0.173	888	0.681	0.496		0.38
	Landscape Richness 1000m	0.140	0.033	0.158	888	0.886	0.376		
	Floral Richness	0.043	0.010	0.121	888	0.353	0.724		
	Floral Density	0.074	0.014	0.133	888	0.559	0.576		
	Species Richness	-0.344	-0.075	0.200	888	-1.725	0.085		
	Apis mellifera Abundance	-0.152	-0.030	0.137	888	-1.107	0.268		
DWV	BQCV	**0.199**	**0.199**	NA	888	6.047	**<0.001**	***	
BQCV	SBV	**0.161**	**0.161**	NA	888	4.856	**<0.001**	***	
SBV	DWV	**0.182**	**0.182**	NA	888	5.504	**<0.001**	***	
Apis mellifera Abundance	Species Richness	**-0.564**	**-0.564**	NA	13	-2.158	**0.028**	*	

Table S8: The full path model statistics for the path model that included *Bombus impatiens* abundance. The table shows the unstandardized estimated pathway coefficients, range-standardized estimated coefficients, standard error, degrees of freedom, critical value, and p-value for each path. R^2 is shown for each response variable. The last four rows are correlated errors. Significant pathway coefficients and p-values are bolded, with negative coefficients in red and positive coefficients in black.

Response variable	Predictor variable	Estimate	Range Std Estimate	Std Error	DF	Critical Value	P-value		R^2
Species Richness	Prop. Natural Area 1000m	**0.633**	**0.910**	0.199	8	3.173	**0.013**	*	0.70
	Landscape Richness 1000m	0.403	0.438	0.241	8	1.673	0.133		
	Floral Richness	0.167	0.179	0.256	8	0.653	0.532		
	Floral Density	-0.236	-0.209	0.278	8	-0.851	0.419		
Bombus impatiens Abundance	Prop. Natural Area 1000m	**0.594**	**0.913**	0.196	8	3.025	**0.016**	*	0.69
	Landscape Richness 1000m	0.431	0.501	0.237	8	1.817	0.107		
	Floral Richness	-0.045	-0.051	0.252	8	-0.178	0.863		
	Floral Density	-0.022	-0.021	0.274	8	-0.081	0.937		
DWV	Prop. Natural Area 1000m	0.043	0.017	0.219	888	0.196	0.844		0.22
	Landscape Richness 1000m	-0.310	-0.093	0.195	888	-1.589	0.112		
	Floral Richness	0.285	0.084	0.175	888	1.621	0.105		
	Floral Density	-0.117	-0.029	0.192	888	-0.609	0.543		
	Species Richness	**-0.602**	**-0.167**	0.273	888	-2.206	**0.027**	*	
	Bombus impatiens Abundance	0.310	0.080	0.273	888	1.136	0.256		
BQCV	Prop. Natural Area 1000m	**0.449**	**0.150**	0.176	888	2.551	**0.011**	*	0.41
	Landscape Richness 1000m	**0.334**	**0.084**	0.149	888	2.247	**0.025**	*	
	Floral Richness	0.250	0.062	0.132	888	1.897	0.058		
	Floral Density	**-0.421**	**-0.086**	0.143	888	-2.942	**0.003**	**	
	Species Richness	**-0.647**	**-0.150**	0.214	888	-3.022	**0.003**	**	
	Bombus impatiens Abundance	-0.076	-0.017	0.202	888	-0.376	0.707		
SBV	Prop. Natural Area 1000m	-0.068	-0.021	0.165	888	-0.410	0.682		0.38
	Landscape Richness 1000m	-0.028	-0.007	0.160	888	-0.176	0.860		
	Floral Richness	0.071	0.017	0.132	888	0.541	0.589		
	Floral Density	0.033	0.006	0.145	888	0.229	0.819		
	Species Richness	-0.391	-0.086	0.211	888	-1.854	0.064		
	Bombus impatiens Abundance	0.321	0.066	0.211	888	1.518	0.129		
DWV	BQCV	**0.199**	**0.199**	NA	888	6.036	**<0.001**	***	
BQCV	SBV	**0.165**	**0.165**	NA	888	4.971	**<0.001**	***	
SBV	DWV	**0.180**	**0.180**	NA	888	5.432	**<0.001**	***	
Bombus impatiens Abundance	Species Richness	**0.510**	**0.510**	NA	13	1.875	**0.045**	*	

Table S9: The full path model statistics for the path model that included *Lasioglossum* spp. abundance. The table shows the unstandardized estimated pathway coefficients, range-standardized estimated coefficients, standard error, degrees of freedom, critical value, and p-value for each path. R^2 is shown for each response variable. The last four rows are correlated errors. Significant pathway coefficients and p-values are bolded, with negative coefficients in red and positive coefficients in black.

Response variable	Predictor variable	Estimate	Range Std Estimate	Std Error	DF	Critical Value	P-value		R^2
Species Richness	Prop. Natural Area 1000m	**0.633**	**0.910**	0.199	8	3.173	**0.013**	*	0.70
	Landscape Richness 1000m	0.403	0.438	0.241	8	1.673	0.133		
	Floral Richness	0.167	0.179	0.256	8	0.653	0.532		
	Floral Density	-0.236	-0.209	0.278	8	-0.851	0.419		
Lasioglossum spp. Abundance	Prop. Natural Area 1000m	0.579	0.818	0.254	8	2.277	0.052		0.43
	Landscape Richness 1000m	0.020	0.021	0.307	8	0.064	0.950		
	Floral Richness	-0.447	-0.471	0.326	8	-1.370	0.208		
	Floral Density	0.103	0.090	0.354	8	0.292	0.778		
DWV	Prop. Natural Area 1000m	0.205	0.082	0.290	888	0.706	0.480		0.22
	Landscape Richness 1000m	-0.221	-0.066	0.201	888	-1.100	0.272		
	Floral Richness	0.213	0.063	0.198	888	1.071	0.284		
	Floral Density	-0.089	-0.022	0.199	888	-0.449	0.653		
	Species Richness	-0.484	-0.134	0.269	888	-1.797	0.072		
	Lasioglossum spp. Abundance	-0.081	-0.023	0.213	888	-0.381	0.703		
BQCV	Prop. Natural Area 1000m	**0.513**	**0.171**	0.202	888	2.540	**0.011**	*	0.41
	Landscape Richness 1000m	**0.340**	**0.086**	0.142	888	2.390	**0.017**	*	
	Floral Richness	0.220	0.055	0.137	888	1.604	0.109		
	Floral Density	**-0.429**	**-0.088**	0.137	888	-3.131	**0.002**	**	
	Species Richness	**-0.732**	**-0.170**	0.188	888	-3.887	**<0.001**	***	
	Lasioglossum spp. Abundance	-0.113	-0.027	0.144	888	-0.781	0.435		
SBV	Prop. Natural Area 1000m	0.046	0.015	0.204	888	0.227	0.821		0.38
	Landscape Richness 1000m	0.057	0.014	0.154	888	0.369	0.712		
	Floral Richness	0.025	0.006	0.140	888	0.180	0.857		
	Floral Density	0.058	0.011	0.142	888	0.412	0.680		
	Species Richness	-0.237	-0.052	0.197	888	-1.205	0.228		
	Lasioglossum spp. Abundance	-0.037	-0.008	0.156	888	-0.239	0.811		
DWV	BQCV	**0.199**	**0.199**	NA	888	6.027	**<0.001**	***	
BQCV	SBV	**0.163**	**0.163**	NA	888	4.899	**<0.001**	***	
SBV	DWV	**0.181**	**0.181**	NA	888	5.468	**<0.001**	***	
Lasioglossum spp. Abundance	Species Richness	-0.435	-0.435	NA	13	-1.530	0.079		

Table S10: The full path model statistics for the path model that included *Eucera pruinosa* abundance. The table shows the unstandardized estimated pathway coefficients, range-standardized estimated coefficients, standard error, degrees of freedom, critical value, and p-value for each path. R^2 is shown for each response variable. The last four rows are correlated errors. Significant pathway coefficients and p-values are bolded, with negative coefficients in red and positive coefficients in black.

Response variable	Predictor variable	Estimate	Range Std Estimate	Std Error	DF	Critical Value	P-value		R^2
Species Richness	Prop. Natural Area 1000m	**0.633**	**0.910**	0.199	8	3.173	**0.013**	*	0.70
	Landscape Richness 1000m	0.403	0.438	0.241	8	1.673	0.133		
	Floral Richness	0.167	0.179	0.256	8	0.653	0.532		
	Floral Density	-0.236	-0.209	0.278	8	-0.851	0.419		
Eucera pruinosa Abundance	Prop. Natural Area 1000m	-0.164	-0.244	0.274	8	-0.600	0.565		0.41
	Landscape Richness 1000m	-0.552	-0.619	0.331	8	-1.667	0.134		
	Floral Richness	0.082	0.091	0.352	8	0.233	0.821		
	Floral Density	0.731	0.664	0.382	8	1.911	0.092		
DWV	Prop. Natural Area 1000m	0.093	0.037	0.207	888	0.448	0.654		0.22
	Landscape Richness 1000m	-0.348	-0.105	0.208	888	-1.672	0.094		
	Floral Richness	0.253	0.075	0.172	888	1.468	0.142		
	Floral Density	0.055	0.013	0.228	888	0.241	0.810		
	Species Richness	-0.438	-0.121	0.232	888	-1.886	0.059		
	Eucera pruinosa Abundance	-0.198	-0.053	0.176	888	-1.126	0.260		
BQCV	Prop. Natural Area 1000m	**0.463**	**0.154**	0.170	888	2.722	**0.006**	**	0.41
	Landscape Richness 1000m	**0.399**	**0.101**	0.162	888	2.458	**0.014**	*	
	Floral Richness	**0.263**	**0.065**	0.129	888	2.037	**0.042**	*	
	Floral Density	**-0.539**	**-0.110**	0.174	888	-3.099	**0.002**	**	
	Species Richness	**-0.703**	**-0.163**	0.181	888	-3.875	**<0.001**	***	
	Eucera pruinosa Abundance	0.151	0.034	0.141	888	1.067	0.286		
SBV	Prop. Natural Area 1000m	-0.038	-0.012	0.144	888	-0.261	0.794		0.38
	Landscape Richness 1000m	-0.058	-0.014	0.150	888	-0.386	0.700		
	Floral Richness	0.040	0.009	0.118	888	0.335	0.738		
	Floral Density	0.222	0.043	0.163	888	1.362	0.173		
	Species Richness	-0.219	-0.048	0.164	888	-1.337	0.181		
	Eucera pruinosa Abundance	-0.234	-0.050	0.127	888	-1.835	0.067		
DWV	BQCV	**0.201**	**0.201**	NA	888	6.097	**<0.001**	***	
BQCV	SBV	**0.166**	**0.166**	NA	888	5.016	**<0.001**	***	
SBV	DWV	**0.176**	**0.176**	NA	888	5.319	**<0.001**	***	
Eucera pruinosa Abundance	Species Richness	-0.037	-0.037	NA	13	-0.117	0.454		

Table S11: The path model with estimated species richness replacing the observed species richness. In this model, species richness was estimated for each site based on a rarefaction curve at the average number of individuals collected across all sites (338 individuals). The unstandardized estimated pathway coefficients, range standardized estimated pathway coefficients, standard error, degrees of freedom, critical value, and p-value for each path. R^2 for each component model. The last four rows are correlated errors included in the model. Significant pathway coefficients and p-values are bolded, with negative coefficients in red and positive coefficients in black. Model AIC was 2516.01, with a ΔAIC of 9.11 compared to the lowest AIC model in **Appendix S2: Table S2**.

Response variable	Predictor variable	Estimate	Range Std Estimate	Std Error	DF	Critical Value	P-value		R^2
Estimated Species Richness	Prop. Natural Area 1000m	**0.547**	**0.799**	0.230	8	2.382	**0.044**	*	0.63
	Landscape Richness 1000m	0.335	0.370	0.278	8	1.207	0.262		
	Floral Richness	0.343	0.373	0.295	8	1.162	0.279		
	Floral Density	-0.251	-0.224	0.320	8	-0.783	0.456		
Apis + *Bombus* Abundance	Prop. Natural Area 1000m	**0.567**	**0.834**	0.186	8	3.049	**0.016**	*	0.71
	Landscape Richness 1000m	0.439	0.488	0.225	8	1.954	0.086		
	Floral Richness	-0.094	-0.103	0.239	8	-0.395	0.703		
	Floral Density	0.084	0.076	0.259	8	0.323	0.755		
DWV	Prop. Natural Area 1000m	-0.087	-0.034	0.250	888	-0.347	0.729		0.22
	Landscape Richness 1000m	-0.389	-0.117	0.218	888	-1.787	0.074		
	Floral Richness	0.315	0.094	0.189	888	1.670	0.095		
	Floral Density	-0.091	-0.022	0.197	888	-0.463	0.644		
	Estimated Species Richness	-0.358	-0.098	0.213	888	-1.683	0.092		
	Apis + *Bombus* Abundance	0.212	0.058	0.260	888	0.816	0.414		
BQCV	Prop. Natural Area 1000m	**0.464**	**0.155**	0.201	888	2.315	**0.021**	*	0.42
	Landscape Richness 1000m	**0.344**	**0.087**	0.170	888	2.021	**0.043**	*	
	Floral Richness	**0.287**	**0.072**	0.145	888	1.983	**0.047**	*	
	Floral Density	**-0.369**	**-0.076**	0.150	888	-2.457	**0.014**	*	
	Estimated Species Richness	**-0.532**	**-0.122**	0.173	888	-3.075	**0.002**	**	
	Apis + *Bombus* Abundance	-0.291	-0.066	0.196	888	-1.483	0.138		
SBV	Prop. Natural Area 1000m	-0.111	-0.035	0.195	888	-0.568	0.570		0.39
	Landscape Richness 1000m	-0.067	-0.016	0.183	888	-0.365	0.715		
	Floral Richness	0.047	0.011	0.146	888	0.322	0.747		
	Floral Density	0.083	0.016	0.152	888	0.543	0.587		
	Estimated Species Richness	-0.121	-0.026	0.172	888	-0.704	0.481		
	Apis + *Bombus* Abundance	0.115	0.025	0.213	888	0.540	0.589		
DWV	BQCV	**0.200**	**0.200**	NA	888	6.074	**<0.001**	***	
BQCV	SBV	**0.168**	**0.168**	NA	888	5.059	**<0.001**	***	
SBV	DWV	**0.181**	**0.181**	NA	888	5.486	**<0.001**	***	
Apis + *Bombus* Abundance	Estimated Species Richness	-0.141	-0.141	NA	13	-0.449	0.331		

References

Dormann, C. F., J. Elith, S. Bacher, C. Buchmann, G. Carl, G. Carré, J. R. García Marquéz, B. Gruber, B. Lafourcade, P. J. Leitão, T. Münkemüller, C. McClean, P. E. Osborne, B. Reineking, B. Schröder, A. K. Skidmore, D. Zurell, and S. Lautenbach. 2013. Collinearity: a review of methods to deal with it and a simulation study evaluating their performance. Ecography 36:27–46.

Fearon, M. L., and E. A. Tibbetts. 2021. Pollinator community species richness dilutes prevalence of multiple viruses within multiple host species. Ecology 102:e03305.

Gotelli, N. J., and R. K. Colwell. 2001. Quantifying biodiversity: Procedures and pitfalls in the measurement and comparison of species richness. Ecology Letters 4:379–391.

Grace, J. B. 2020. A “weight of evidence” approach to evaluating structural equation models. One Ecosystem 5:e50452.

Hartig, F. 2020. DHARMa: Residual Diagnostics for Hierarchical (Multi-Level/Mixed) Regression Models. https://cran.r-project.org/package=DHARMa.

Hebbali, A. 2020. olsrr: Tools for Building OLS Regression Models.

Hsieh, T. C., K. H. Ma, and A. Chao. 2016. iNEXT: an R package for rarefaction and extrapolation of species diversity (Hill numbers). Methods in Ecology and Evolution 7:1451–1456.

Lefcheck, J. S. 2016. PIECEWISE SEM: Piecewise structural equation modelling in R for ecology, evolution, and systematics. Methods in Ecology and Evolution 7:573–579.

Novotný, V., and Y. Basset. 2000. Rare species in communities of tropical insect herbivores: Pondering the mystery of singletons. Oikos 89:564–572.

Paradis, E., and K. Schliep. 2018. ape 5.0: an environment for modern phylogenetics and evolutionary analyses in R. Bioinformatics 35:526–528.

R Core Team. 2021. R: A language and environment for statistical computing. R Foundation for Statistical Computing, Vienna, Austria.
