# Supplementary material for: Habitat quality influences pollinator pathogen prevalence through both habitat–disease and biodiversity–disease pathways
Source: Ecology. 2023 Jan 3;104(2):e3933. doi: 10.1002/ecy.3933 (PMC10078577; doi:10.1002/ecy.3933)
Supplement: Supplementary file 3 — Appendix S3. [file ECY-104-0-s003.pdf]

Appendix S3

Manuscript title: Habitat Quality Influences Pollinator Pathogen Prevalence Through Both Habitat–Disease and Biodiversity–Disease Pathways

Authors: Michelle L. Fearon, Chelsea L. Wood, Elizabeth A. Tibbetts

Journal: Ecology

Section S1: Additional details about the host species-specific path models

We also conducted species-specific path models for *A. mellifera*, *B. impatiens*, *Lasioglossum* spp., and *E. pruinosa* to be able to assess whether habitat or pollinator community variables impacted viral prevalence within each host species differently (i.e., the full dataset was subset to only include viral presence/absence data for a single host species). For each of the species-specific path models, we started with the same initial path structure as the main model with total pollinator abundance described in the main text and **Appendix S2**. However, we also compared path models where we substituted the total pollinator abundance for either *A. mellifera*, *B. impatiens*, combined *A. mellifera* and *B. impatiens*, or the species-specific abundance if not already included in the list above. For all four species, the models that included combined *A. mellifera* and *B. impatiens* abundance had the lowest AIC (AIC and detailed model output tables for models not presented are available in associated R code files [https://doi.org/10.5061/dryad.mkkwh710c]). Therefore, we selected the species-specific models that included the combined *A. mellifera* and *B. impatiens* abundance as the models to present in the main text (**Figure 3; Appendix S3: Tables S1-S4**), which also allows for easier comparison to the main path model presented (**Figure 1b**).

All species-specific path models were fully saturated; therefore, we were unable to conduct chi-squared and d-separation tests to assess goodness of fit for the full models. We used the same temporary model simplification approach as described in **Appendix S2: Section S1** for each of the top species-specific models to ensure good model fit with our data by removing paths with p-values greater than 0.8. The simplified species-specific models all fit the data well as shown by the goodness of fit tests (*Apis*: χ^2=0.277, p=0.999, Fisher's C=1.636; p=0.998; *Bombus*: χ^2=0.009, p=0.924, Fisher's C=0.159; p=0.924; *Lasioglossum*: χ^2=0.001, p=0.98, Fisher's C=0.04; p=0.98; *Eucera*: χ^2=0.042, p=0.979; Fisher's C=0.406; p=0.982;). The full output for each species' simplified model can be seen in the manuscript associated code. All models shown in the main text include all paths.

Table S1: Path model for habitat quality and pollinator community factors effects on viral prevalence in *Apis mellifera* only (**Figure 3a**). The unstandardized estimated pathway coefficients, range standardized estimated pathway coefficients, standard error, degrees of freedom, critical value, and p-value for each path. R^2 for each component model. The last four rows are correlated errors included in the model. Significant pathway coefficients and p-values are bolded, with negative coefficients in red and positive coefficients in black.

Response variable	Predictor variable	Estimate	Range Std Estimate	Std Error	DF	Critical Value	P-value		R^2
Species Richness	Prop. Natural Area 1000m	**0.633**	**0.910**	0.199	8	3.173	**0.013**	*	0.70
	Landscape Richness 1000m	0.403	0.438	0.241	8	1.673	0.133		
	Floral Richness	0.167	0.179	0.256	8	0.653	0.532		
	Floral Density	-0.236	-0.209	0.278	8	-0.851	0.419		
Apis + Bombus Abundance	Prop. Natural Area 1000m	**0.567**	**0.834**	0.186	8	3.049	**0.016**	*	0.71
	Landscape Richness 1000m	0.439	0.488	0.225	8	1.954	0.086		
	Floral Richness	-0.094	-0.103	0.239	8	-0.395	0.703		
	Floral Density	0.084	0.076	0.259	8	0.323	0.755		
DWV	Prop. Natural Area 1000m	-0.369	-0.158	0.397	234	-0.931	0.352		0.16
	Landscape Richness 1000m	-0.561	-0.181	0.336	234	-1.669	0.095		
	Floral Richness	0.187	0.059	0.274	234	0.681	0.496		
	Floral Density	0.007	0.002	0.293	234	0.023	0.982		
	Species Richness	-0.294	-0.087	0.382	234	-0.770	0.441		
	Apis + Bombus Abundance	0.495	0.144	0.408	234	1.213	0.225		
BQCV	Prop. Natural Area 1000m	-0.037	-0.017	0.287	234	-0.130	0.897		0.02
	Landscape Richness 1000m	0.267	0.092	0.240	234	1.110	0.267		
	Floral Richness	0.198	0.067	0.194	234	1.020	0.308		
	Floral Density	-0.276	-0.077	0.210	234	-1.311	0.190		
	Species Richness	-0.267	-0.084	0.293	234	-0.911	0.362		
	Apis + Bombus Abundance	-0.073	-0.022	0.302	234	-0.241	0.810		
SBV	Prop. Natural Area 1000m	-0.054	-0.025	0.277	234	-0.194	0.846		0.04
	Landscape Richness 1000m	0.075	0.026	0.258	234	0.289	0.772		
	Floral Richness	-0.018	-0.006	0.208	234	-0.088	0.930		
	Floral Density	0.199	0.055	0.223	234	0.889	0.374		
	Species Richness	-0.177	-0.056	0.294	234	-0.601	0.548		
	Apis + Bombus Abundance	-0.141	-0.044	0.324	234	-0.436	0.663		
DWV	BQCV	**0.119**	**0.119**	NA	234	1.829	**0.034**	*	
BQCV	SBV	**0.193**	**0.193**	NA	234	2.996	**0.002**	**	
SBV	DWV	**0.193**	**0.193**	NA	234	2.986	**0.002**	**	
Apis + Bombus Abundance	Species Richness	0.149	0.149	NA	13	0.475	0.323		

Table S2: Path model for habitat quality and pollinator community factors effects on viral prevalence in *Bombus impatiens* only (**Figure 3b**). The unstandardized estimated pathway coefficients, range standardized estimated pathway coefficients, standard error, degrees of freedom, critical value, and p-value for each path. R^2 for each component model. The last four rows are correlated errors included in the model. Significant pathway coefficients and p-values are bolded, with negative coefficients in red and positive coefficients in black.

Response variable	Predictor variable	Estimate	Range Std Estimate	Std Error	DF	Critical Value	P-value	R^2
Species Richness	Prop. Natural Area 1000m	**0.633**	**0.910**	0.199	8	3.173	**0.013** *	0.70
	Landscape Richness 1000m	0.403	0.438	0.241	8	1.673	0.133	
	Floral Richness	0.167	0.179	0.256	8	0.653	0.532	
	Floral Density	-0.236	-0.209	0.278	8	-0.851	0.419	
Apis + *Bombus* Abundance	Prop. Natural Area 1000m	**0.567**	**0.834**	0.186	8	3.049	**0.016** *	0.71
	Landscape Richness 1000m	0.439	0.488	0.225	8	1.954	0.086	
	Floral Richness	-0.094	-0.103	0.239	8	-0.395	0.703	
	Floral Density	0.084	0.076	0.259	8	0.323	0.755	
DWV	Prop. Natural Area 1000m	-0.095	-0.041	0.353	248	-0.268	0.789	0.13
	Landscape Richness 1000m	-0.290	-0.095	0.304	248	-0.955	0.339	
	Floral Richness	**0.571**	**0.184**	0.249	248	2.295	**0.022** *	
	Floral Density	0.026	0.007	0.269	248	0.096	0.924	
	Species Richness	-0.158	-0.047	0.337	248	-0.468	0.640	
	Apis + *Bombus* Abundance	0.142	0.042	0.351	248	0.405	0.685	
BQCV	Prop. Natural Area 1000m	**0.826**	**0.350**	0.261	248	3.165	**0.002** **	0.13
	Landscape Richness 1000m	0.306	0.098	0.214	248	1.433	0.152	
	Floral Richness	**0.716**	**0.226**	0.224	248	3.204	**0.001** **	
	Floral Density	**-0.795**	**-0.207**	0.267	248	-2.981	**0.003** **	
	Species Richness	**-0.702**	**-0.207**	0.257	248	-2.730	**0.006** **	
	Apis + *Bombus* Abundance	-0.188	-0.054	0.250	248	-0.754	0.451	
SBV	Prop. Natural Area 1000m	0.098	0.045	0.259	248	0.377	0.706	0.04
	Landscape Richness 1000m	0.091	0.031	0.222	248	0.408	0.683	
	Floral Richness	0.288	0.098	0.188	248	1.526	0.127	
	Floral Density	-0.258	-0.072	0.205	248	-1.260	0.208	
	Species Richness	-0.430	-0.136	0.254	248	-1.691	0.091	
	Apis + *Bombus* Abundance	0.250	0.077	0.253	248	0.985	0.325	
DWV	BQCV	**0.296**	**0.296**	NA	248	4.853	**<0.001** ***	
BQCV	SBV	**0.172**	**0.172**	NA	248	2.729	**0.003** **	
SBV	DWV	**0.210**	**0.210**	NA	248	3.354	**<0.001** ***	
Apis + *Bombus* Abundance	Species Richness	0.149	0.149	NA	13	0.475	0.323	

Table S3: Path model for habitat quality and pollinator community factors effects on viral prevalence in *Lasioglossum* spp. only (**Figure 3c**). The unstandardized estimated pathway coefficients, range standardized estimated pathway coefficients, standard error, degrees of freedom, critical value, and p-value for each path. R^2 for each component model. The last four rows are correlated errors included in the model. Significant pathway coefficients and p-values are bolded, with negative coefficients in red and positive coefficients in black.

Response variable	Predictor variable	Estimate	Range Std Estimate	Std Error	DF	Critical Value	P-value		R^2
Species Richness	Prop. Natural Area 1000m	**0.633**	**0.910**	0.199	8	3.173	**0.013**	*	0.70
	Landscape Richness 1000m	0.403	0.438	0.241	8	1.673	0.133		
	Floral Richness	0.167	0.179	0.256	8	0.653	0.532		
	Floral Density	-0.236	-0.209	0.278	8	-0.851	0.419		
Apis + *Bombus* Abundance	Prop. Natural Area 1000m	**0.567**	**0.834**	0.186	8	3.049	**0.016**	*	0.71
	Landscape Richness 1000m	0.439	0.488	0.225	8	1.954	0.086		
	Floral Richness	-0.094	-0.103	0.239	8	-0.395	0.703		
	Floral Density	0.084	0.076	0.259	8	0.323	0.755		
DWV	Prop. Natural Area 1000m	-0.376	-0.148	0.420	233	-0.895	0.371		0.14
	Landscape Richness 1000m	**-0.758**	**-0.225**	0.351	233	-2.162	**0.031**	*	
	Floral Richness	**0.942**	**0.276**	0.368	233	2.563	**0.010**	*	
	Floral Density	-0.564	-0.136	0.503	233	-1.120	0.263		
	Species Richness	**-0.793**	**-0.217**	0.378	233	-2.097	**0.036**	*	
	Apis + *Bombus* Abundance	0.629	0.168	0.443	233	1.419	0.156		
BQCV	Prop. Natural Area 1000m	1.714	0.631	0.956	233	1.793	0.073		0.09
	Landscape Richness 1000m	0.619	0.172	0.721	233	0.859	0.390		
	Floral Richness	-0.015	-0.004	0.616	233	-0.025	0.980		
	Floral Density	-0.402	-0.091	0.777	233	-0.517	0.605		
	Species Richness	**-2.109**	**-0.540**	0.891	233	-2.369	**0.018**	*	
	Apis + *Bombus* Abundance	0.256	0.064	0.622	233	0.412	0.680		
DWV	BQCV	0.052	0.052	NA	233	0.786	0.216		
Apis + *Bombus* Abundance	Species Richness	0.149	0.149	NA	13	0.475	0.323		

Table S4: Path model for habitat quality and pollinator community factors effects on viral prevalence in *Eucera pruinosa* only (**Figure 3d**). Note that one field site did not contain any *E. pruinosa* individuals and therefore that site was excluded from this analysis. As a result, the pathway coefficients for the site-level links between habitat factors and pollinator species richness and abundance are slightly altered compared to the other species-specific path models. The unstandardized estimated pathway coefficients, range standardized estimated pathway coefficients, standard error, degrees of freedom, critical value, and p-value for each path. R^2 for each component model. The last four rows are correlated errors included in the model. Significant pathway coefficients and p-values are bolded, with negative coefficients in red and positive coefficients in black.

Response variable	Predictor variable	Estimate	Range Std Estimate	Std Error	DF	Critical Value	P-value		R^2
Species Richness	Prop. Natural Area 1000m	**0.601**	**0.864**	0.191	7	3.156	**0.016**	*	0.75
	Landscape Richness 1000m	0.432	0.470	0.229	7	1.885	0.101		
	Floral Richness	0.183	0.196	0.243	7	0.751	0.477		
	Floral Density	-0.234	-0.206	0.264	7	-0.888	0.404		
Apis + Bombus Abundance	Prop. Natural Area 1000m	**0.581**	**0.854**	0.196	7	2.967	**0.021**	*	0.72
	Landscape Richness 1000m	0.426	0.474	0.236	7	1.809	0.113		
	Floral Richness	-0.101	-0.111	0.250	7	-0.405	0.698		
	Floral Density	0.083	0.075	0.271	7	0.306	0.769		
DWV	Prop. Natural Area 1000m	**0.929**	**0.387**	0.352	173	2.641	**0.008**	**	0.10
	Landscape Richness 1000m	-0.135	-0.042	0.274	173	-0.493	0.622		
	Floral Richness	-0.012	-0.004	0.329	173	-0.036	0.972		
	Floral Density	-0.288	-0.074	0.409	173	-0.703	0.482		
	Species Richness	-0.781	-0.226	0.404	173	-1.934	0.053		
	Apis + Bombus Abundance	-0.108	-0.031	0.419	173	-0.258	0.796		
BQCV	Prop. Natural Area 1000m	0.244	0.104	0.711	173	0.342	0.732		0.06
	Landscape Richness 1000m	0.594	0.193	0.627	173	0.947	0.344		
	Floral Richness	-0.351	-0.112	0.434	173	-0.808	0.419		
	Floral Density	-0.084	-0.022	0.416	173	-0.201	0.840		
	Species Richness	-0.625	-0.186	0.672	173	-0.929	0.353		
	Apis + Bombus Abundance	0.411	0.120	0.710	173	0.579	0.563		
DWV	BQCV	**0.249**	**0.249**	NA	173	3.354	**0.0005**	***	
Apis + Bombus Abundance	Species Richness	0.284	0.284	NA	12	0.890	0.198		
